# Functionalized GelMA-based hydrogels for osteoimmunomodulation and bone regeneration

**DOI:** 10.3389/fbioe.2026.1969286

**Published:** 2026-09-23

**Authors:** Li Chen, Jingyao Tan, Maolin Li, Ying Xia, Yan Huang, Xiaoyi Liu, Jie Gao, Juan Zeng, Kun Yang

**Affiliations:** Department of Periodontology, The Affiliated Stomatological Hospital of Zunyi Medical University, Zunyi, China

**Keywords:** bone regeneration, functionalized hydrogels, gelatin methacryloyl, osteoimmune microenvironment, osteoimmunomodulation, tissue engineering

## Abstract

Complex bone defects caused by trauma, infection, tumor-related bone loss and metabolic disorders remain clinically challenging because an inflammatory imbalance, impaired vascularization and insufficient osteogenesis often coexist. Gelatin methacryloyl (GelMA) hydrogels are attractive matrices for bone regeneration owing to the extracellular matrix-mimicking structure, tunable physicochemical properties and compatibility with injectable, printable and composite systems. However, pristine GelMA lacks the mechanical strength, osteoinductivity and immunoregulatory capacity required for complex bone repair. This review summarizes recent advances in functionalized GelMA-based hydrogels for osteoimmunomodulation and bone regeneration. We first outline the immune-regenerative mechanisms of bone repair and then discuss major functionalization strategies, including matrix-property regulation, inorganic nanocomponents, bioactive molecules, extracellular vesicles, blood-derived products, and microenvironment-responsive systems. Their applications in critical-sized, infected, metabolic disorder-associated, and oral and maxillofacial bone defects are further examined from a pathology-adapted and mechanism-guided perspective. Collectively, these systems are transforming GelMA from a passive scaffold into a programmable platform that coordinates inflammation resolution, vascularized osteogenesis, and remodeling. By linking material design with osteoimmune-regenerative mechanisms and disease-specific applications, this review provides a framework relevant to the development and translation of advanced regenerative biomaterials.

## Graphical Abstract

**Figure:**
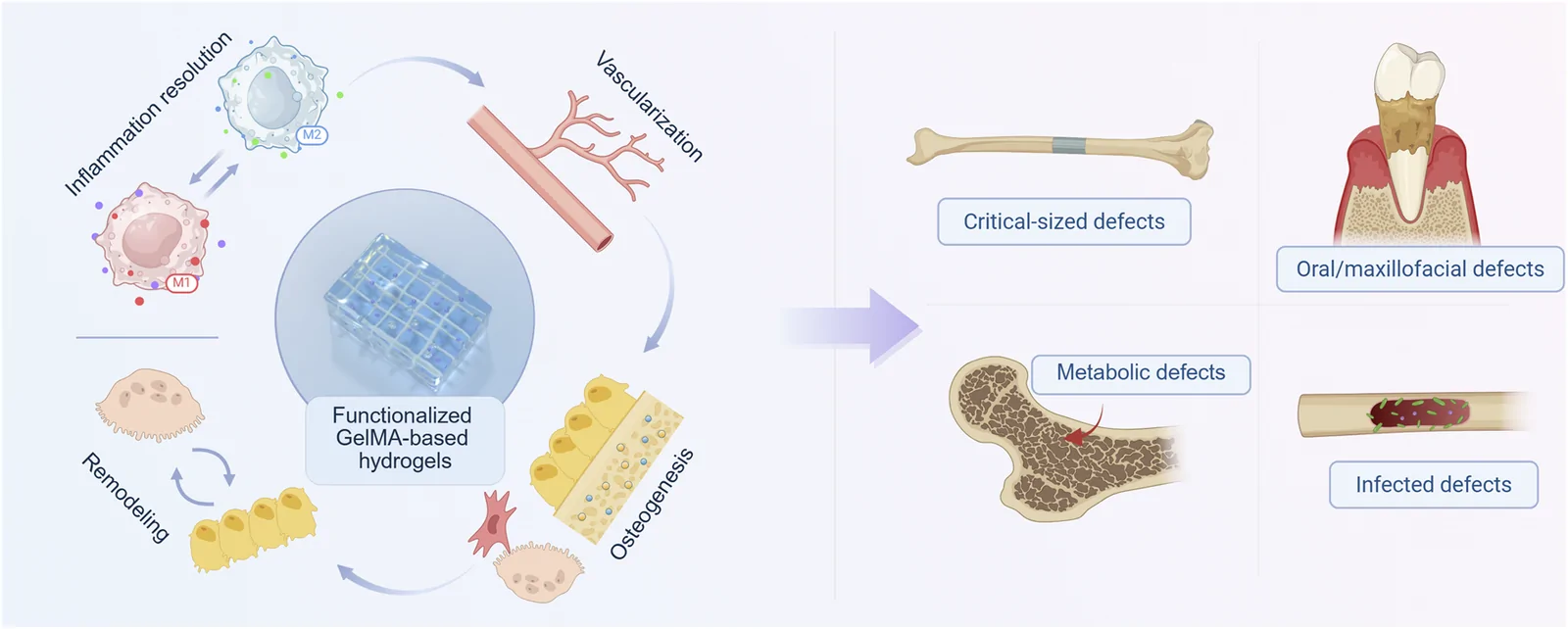
Created in BioRender. Chen, L. (2026) https://BioRender.com/v86tydd.

## 1 Introduction

Bone defects resulting from trauma, infection, tumor resection, and metabolic disorders remain common and clinically challenging conditions in bone repair (Xue et al., 2022). Although bone possesses an intrinsic capacity for self-repair, this capacity is often insufficient for critical-sized defects (Schemitsch, 2017). Repair may be further compromised by infection, chronic inflammation, and metabolic dysregulation, which disrupt the local regenerative microenvironment (Newman et al., 2021; Sheng et al., 2023; Zhang H. et al., 2025). Bone grafting remains a mainstay of defect reconstruction (Baldwin et al., 2019; Schmidt, 2021). However, autologous grafts are limited by donor-site morbidity and restricted availability (Schmidt, 2021), whereas allogeneic grafts and synthetic substitutes exhibit variable regenerative efficacy and context-dependent clinical limitations (Baldwin et al., 2019). These shortcomings underscore the need for bone-regenerative biomaterials that not only fill defects or provide mechanical support but also actively guide local biological responses toward coordinated tissue repair.

Bone regeneration is a temporally coordinated process involving inflammation, angiogenesis, osteogenesis, and remodeling (Molitoris et al., 2024; Xu et al., 2024). Rather than serving solely as a defensive response after injury or implantation, the immune response acts as a key upstream regulator that influences whether the local microenvironment progresses toward regeneration, persistent inflammation or fibrotic encapsulation (Martin and García, 2021). In particular, macrophage plasticity and phenotypic transitions, immune-cell crosstalk and immune–vascular–osteogenic coupling have emerged as major determinants of bone repair outcomes (Martin and García, 2021; Liu et al., 2025b). Accordingly, the design of bone-regenerative biomaterials has increasingly shifted beyond osteoconductive or osteoinductive support toward osteoimmunomodulation (Lee et al., 2019), in which material properties and bioactive cues are engineered to guide host responses and promote functional tissue reconstruction.

Hydrogels provide versatile platforms for this design concept because their hydrated three-dimensional networks can mimic key features of the extracellular matrix (ECM), while allowing controllable modification of their mechanical, structural, and biochemical properties (Cao et al., 2021). Among them, gelatin methacryloyl (GelMA) has attracted considerable attention owing to its cell-adhesive motifs, matrix metalloproteinase-responsive sequences, photocrosslinkable processability and compatibility with injectable, printable and composite systems (Yue et al., 2015). Nevertheless, pristine GelMA generally lacks the mechanical robustness, intrinsic osteoinductivity, and immunoregulatory capacity required for complex bone repair (Huang et al., 2025). Functionalization is therefore essential to transform GelMA from a primarily structural matrix into a bioactive and osteoimmunomodulatory platform.

Importantly, many functional agents used in GelMA-based systems, including extracellular vesicles, nanozymes, metal–organic frameworks and blood-derived products, can also be incorporated into other hydrogel backbones. Their intrinsic biological activities should therefore be distinguished from the matrix-specific functions of GelMA. GelMA combines gelatin-derived cell-adhesive and matrix metalloproteinase-sensitive motifs with methacryloyl-mediated photocrosslinkability, thereby integrating intrinsic cell–matrix interactions with tunable crosslinking and fabrication (Yue et al., 2015; Bupphathong et al., 2022). Compared with relatively bioinert matrices such as unmodified alginate and pristine PEG, GelMA intrinsically retains gelatin-derived cell-interactive motifs, whereas synthetic matrices such as PEG can offer greater mechanical robustness and physicochemical tunability but generally require additional biofunctionalization to support cell adhesion and cell-mediated remodeling (Cao et al., 2021). Compared with unmodified gelatin, methacryloyl functionalization further enables light-triggered covalent crosslinking and more controllable fabrication while retaining key gelatin-derived biological motifs (Yue et al., 2015). Nevertheless, pristine GelMA remains limited by relatively weak mechanical strength, formulation-dependent physicochemical properties and imperfect control of degradation, particularly for load-bearing bone repair (Huang et al., 2025). Accordingly, the following sections distinguish the bioactivity of incorporated functional components from the matrix-level roles of GelMA in cell–matrix interactions, structural integration, local retention and controllable presentation of therapeutic cues.

From a material-design perspective, functionalized GelMA-based hydrogels can regulate the osteoimmune microenvironment through several interconnected mechanisms. At the matrix level, stiffness, pore architecture, degradation behavior and interfacial properties influence macrophage responses, cell and tissue infiltration, and degradation–regeneration matching (Zhuang et al., 2020; Jiang et al., 2021; Huang et al., 2025). Inorganic components can reinforce GelMA networks while providing ion-mediated antibacterial, redox-regulatory and osteogenic cues (Jiang et al., 2026; Jin et al., 2026). Bioactive molecules, extracellular vesicles and blood-derived products enable the localized and controlled delivery of immunomodulatory, angiogenic and osteogenic signals (Li L. et al., 2026; Zhang X. et al., 2026). Microenvironment-responsive systems can further react to pathological or externally applied stimuli, including acidic pH, excessive reactive oxygen species (ROS), infection-associated signals and light, thereby enabling spatiotemporally controlled regulation of inflammation and tissue repair (Wu et al., 2024; Li C. et al., 2026; Qi et al., 2026; Zhou C. et al., 2026). Collectively, these strategies are transforming GelMA-based hydrogels from passive scaffolds into programmable platforms for coordinating immune regulation, vascularization, and osteogenesis.

Previous reviews have primarily focused on GelMA composite design, nanocomposite construction, infection control, bioink development, or general bone-regenerative performance (Zhu et al., 2024d; Huang et al., 2025; Li G. et al., 2026). However, a framework that systematically connects GelMA functionalization strategies with osteoimmune-regenerative mechanisms and further adapts these mechanisms to distinct pathological bone-defect microenvironments remains lacking. This review therefore establishes a design-oriented framework linking GelMA functionalization, osteoimmune-regenerative mechanisms and pathology-specific repair requirements. We first outline the biological basis of osteoimmune regulation during bone repair and then examine the major functionalization strategies of GelMA-based hydrogels. Their applications are subsequently discussed across critical-sized, infected, metabolic abnormality-associated, and oral and maxillofacial bone defects. Finally, current translational challenges and future directions are considered to guide the development of pathology-adapted and clinically relevant GelMA-based biomaterials for bone regeneration.

## 2 Osteoimmune regulation during bone repair

During bone defect repair, inflammation is not merely a detrimental response but a regulatory process that determines whether regeneration is appropriately initiated and successfully progresses toward repair (Molitoris et al., 2024). After bone injury, vascular disruption and hematoma formation establish a metabolically stressed inflammatory niche characterized by local hypoxia, acidosis, and lactate accumulation (Maruyama et al., 2020). Within this context, necrotic cell debris and damaged extracellular matrix components serve as endogenous sources of damage-associated molecular patterns (DAMPs), while pathogen-associated molecular patterns (PAMPs) may additionally contribute to immune activation in infected or contaminated defects (Maruyama et al., 2020; Ma et al., 2024). Recognition of these molecular cues by pattern-recognition receptors rapidly activates innate immune signaling, leading to the production of inflammatory mediators and the recruitment of immune cells to the defect site (Burgan et al., 2025). Temporally, this process can be viewed as a series of overlapping immune-regenerative windows, including early innate immune activation dominated by inflammatory macrophage responses, a subsequent inflammation-resolution window characterized by macrophage reprogramming toward reparative states, a reparative phase involving pro-angiogenic and osteogenic signaling, and a later remodeling phase governed by osteoblast–osteoclast coupling (Gou et al., 2024; Molitoris et al., 2024; Burgan et al., 2025). Transient and appropriately controlled inflammation facilitates the clearance of necrotic tissue and potential pathogens, while providing chemokines, growth factors and matrix-remodeling signals that support reparative cell recruitment, angiogenesis, and bone formation (Newman et al., 2021; Molitoris et al., 2024). By contrast, excessive or unresolved inflammation may impair vascularization, suppress osteogenesis and enhance bone resorption, thereby driving the defect site toward chronic inflammation, regenerative arrest and, ultimately, delayed healing or non-union (Newman et al., 2021; Menger et al., 2022). The immune-regenerative sequence of bone repair is summarized in Figure 1.

**FIGURE 1:**
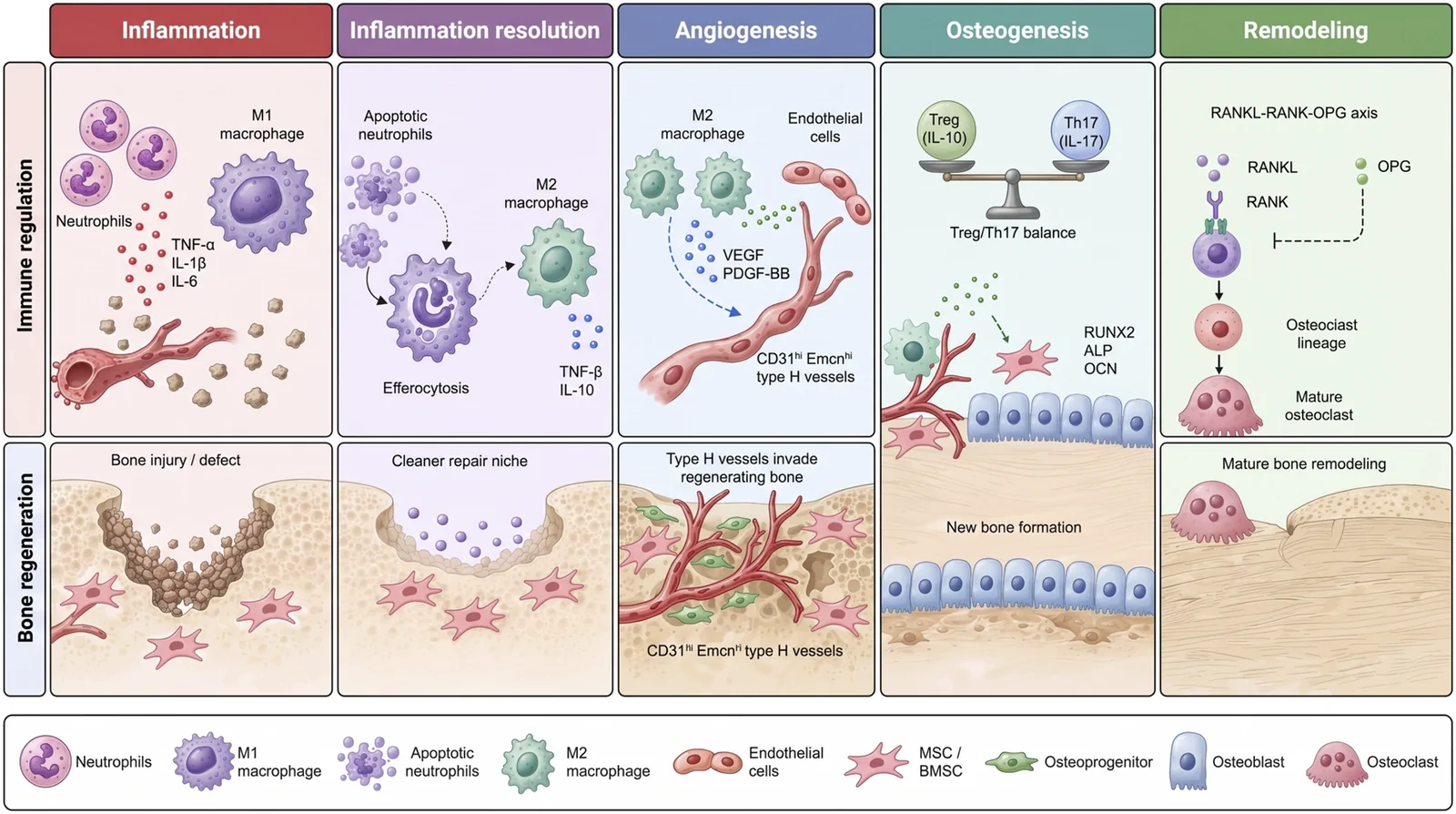
Osteoimmune regulation during bone repair. This schematic summarizes the temporal coordination between immune regulation and bone regeneration during bone repair, including inflammation, inflammation resolution, angiogenesis, osteogenic differentiation, and osteoblast–osteoclast mediated remodeling. Created in BioRender. Chen, L. (2026) https://BioRender.com/2wp48h7.

Immune regulation during bone repair is governed by a multicellular cooperative network, in which dynamic interactions between innate and adaptive immune cells shape the final regenerative outcome (Yang and Liu, 2021). In the early phase of injury, neutrophils are among the first immune cells recruited to the defect site. Through phagocytosis, degranulation, and cytokine–chemokine signaling, they contribute to pathogen and debris clearance and amplify early inflammatory-cell recruitment (Molitoris et al., 2024; Wang R. et al., 2024; Burgan et al., 2025). Neutrophils may also contribute structurally to early repair by depositing a fibronectin-rich provisional ECM within the fracture hematoma, thereby facilitating subsequent stromal-cell infiltration and matrix formation (Bastian et al., 2016). However, under metabolic stress such as diabetes, excessive neutrophil activation and NETosis can lead to aberrant accumulation of neutrophil extracellular traps (NETs), sustaining local inflammation and suppressing osteogenic activity, thereby delaying bone healing (Zhu et al., 2023).

Following this initial defense and signaling phase, macrophages become central regulators of microenvironmental remodeling during bone repair (Gou et al., 2024). Although macrophage activation comprises a continuum of heterogeneous and plastic states rather than discrete M1 and M2 phenotypes, the M1/M2 framework remains useful for interpreting biomaterial-mediated immune regulation (Murray et al., 2014; Xie et al., 2020). In the early inflammatory phase, pro-inflammatory M1 macrophages participate in lesion clearance and chemotactic signal amplification. As repair progresses, macrophages gradually shift along a phenotypic continuum toward anti-inflammatory, pro-angiogenic, and tissue-remodeling states (Gou et al., 2024). Within this continuum, reparative macrophage states may display inflammation-resolving, efferocytic, matrix-remodeling or pro-angiogenic functions, depending on the local signals and stage of repair (Fuchs et al., 2024). Beyond cytokine-mediated polarization, efferocytosis links the macrophage-mediated clearance of apoptotic neutrophils with inflammation resolution and reparative macrophage programming (Miszuk et al., 2025). Efficient efferocytosis limits secondary necrosis and inflammatory amplification, whereas impaired efferocytosis under pathological conditions such as diabetes and periodontitis may delay reparative remodeling and prolong local inflammation (Li B. et al., 2023; Miszuk et al., 2025). Accordingly, biomaterial strategies that enhance macrophage efferocytosis may extend immunomodulation from simple suppression of inflammation toward active resolution of inflammation, as illustrated by recent efferocytosis-targeted approaches for bone repair (Miszuk et al., 2025). Through the secretion of bioactive mediators such as IL-10, transforming growth factor-β (TGF-β) and vascular endothelial growth factor (VEGF), reparative macrophages coordinate inflammation resolution, angiogenic remodeling, and osteogenic differentiation (Gou et al., 2024). Thus, the transition from inflammatory activation toward reparative remodeling, rather than a rigid binary shift between macrophage phenotypes, provides a practical framework for understanding osteoimmune regulation and biomaterial-mediated immunomodulation.

Importantly, innate and adaptive immune responses are not temporally or functionally isolated during bone repair. Cytokines and chemokines released by innate immune cells, together with antigen-presenting signals, influence T-cell recruitment, activation and differentiation, thereby shaping the subsequent balance between pro-inflammatory effector responses and regulatory immune states. Adaptive immune cells, in turn, feed back on macrophages, stromal cells and osteoclast-lineage cells through cytokine and RANKL/OPG-related signaling. This bidirectional crosstalk therefore provides a mechanistic bridge through which early innate immune events can influence later inflammation resolution, bone remodeling and regenerative outcome (Yang and Liu, 2021; Wang R. et al., 2024).

In addition to innate immune cells, adaptive immune cells also regulate the mid-to-late stages of bone repair (Yang and Liu, 2021). T lymphocyte subsets influence the intensity and duration of inflammation and contribute to the regulation of bone remodeling (Wang R. et al., 2024). In bone metabolism, the balance between effector T cells and regulatory T cells (Tregs) is particularly important. Under inflammatory bone-remodeling conditions, Th17 cells can promote osteoclastogenesis and bone resorption mainly through IL-17-mediated enhancement of RANKL signaling, whereas Tregs suppress inflammatory cascades by secreting IL-10 and TGF-β and by interacting with bone marrow mesenchymal stem cells (BMSCs) and other stromal components (Zhu et al., 2024b). Recent studies further indicate that Treg-mediated regulation is disease-specific and injury-responsive; for example, injury-responsive Tregs can directly participate in fracture healing by secreting progranulin (PGRN) (Chen R. et al., 2025). B-cell subsets further contribute to osteoblast–osteoclast balance. Mature B cells can maintain bone homeostasis by producing osteoprotegerin (OPG), a decoy receptor that prevents receptor activator of nuclear factor-κB ligand (RANKL) from binding to RANK, thereby suppressing osteoclast differentiation (Li et al., 2007; Udagawa et al., 2021). Together, these findings indicate that adaptive immune cells participate not only in inflammatory regulation but also in the maintenance of osteogenic–osteoclastic homeostasis. From a biomaterial perspective, these findings imply that osteoimmunomodulation should not be evaluated solely by early macrophage polarization. A more complete assessment should determine whether early innate immune modulation subsequently supports a Treg/Th17 balance and RANKL/OPG environment compatible with controlled osteoclastogenesis and stable bone remodeling (Li et al., 2007; Udagawa et al., 2021; Zhu et al., 2024b).

The importance of the osteoimmune microenvironment in bone regeneration lies in the spatiotemporal coupling of immune regulation with angiogenesis, osteogenic differentiation, and osteoclast-mediated remodeling (Grosso et al., 2017; Yang and Liu, 2021). This coupling is particularly evident during the proliferative and remodeling phases of repair. Infiltrating immune cells and local stromal cells release angiogenic and osteogenic cues, including VEGF and bone morphogenetic proteins (BMPs). VEGF supports endothelial-cell proliferation, migration and angiogenic sprouting through VEGFR-dependent signaling, whereas BMP signaling promotes osteogenic differentiation and contributes to angiogenesis–osteogenesis coupling (Grosso et al., 2017; Li S. et al., 2025). Endothelial cells, in turn, regulate local perfusion and immune-cell trafficking through vasoactive mediator release, adhesion-molecule expression, vascular permeability and leukocyte transmigration (Amersfoort et al., 2022). Beyond endothelial sprouting, reparative M2 macrophages may support vascular maturation by secreting platelet-derived growth factor-BB (PDGF-BB), which promotes PDGFRβ-dependent pericyte recruitment and coverage of nascent vessels. This stabilized vascular network improves oxygen and nutrient supply and facilitates osteoprogenitor delivery, forming a positive feedback loop among immune regulation, vascular maturation and osteogenic initiation. The establishment of this immune-regulated vascular maturation loop is therefore a desirable outcome for bone-regenerative biomaterials, including functionalized GelMA-based systems (Li et al., 2021; Li S. et al., 2025). The identification of type H vessels, characterized by high expression of CD31 and endomucin, further supports the view that functional vascularization is an active component of the osteogenic microenvironment rather than a passive consequence of bone formation (Kusumbe et al., 2014).

Bone formation and bone resorption are subsequently coordinated through immune–osteogenic–osteoclastic feedback loops (Tsukasaki and Takayanagi, 2019). Excessive or persistent pro-inflammatory signaling may suppress osteogenic differentiation by activating NF-κB-related inflammatory pathways and interfering with Wnt/β-catenin signaling, thereby reducing osteogenic marker expression and impairing osteoblast-lineage maturation (Chang et al., 2013; Zhu et al., 2024a). In parallel, Th17/IL-17-driven inflammation can promote osteoclast-mediated bone resorption by increasing RANKL expression (Zhu et al., 2024b). Conversely, osteoclast-mediated bone matrix degradation releases and activates matrix-embedded growth factors, such as TGF-β and IGF-1, which recruit MSCs to resorption sites and regulate osteogenic differentiation during coupled bone remodeling (Xu et al., 2018). The RANKL–RANK–OPG axis is central to this process because it regulates osteoclast differentiation, survival and resorptive activity, thereby influencing the balance between bone formation and resorption (Udagawa et al., 2021; Grewe et al., 2022). Therefore, successful regeneration requires not only osteogenic induction but also coordinated control of osteoclast activity and long-term remodeling.

Under pathological conditions, however, this temporally coordinated coupling can be disrupted. In diabetes, persistent hyperglycemia and chronic low-grade inflammation impair macrophage plasticity and hinder the timely transition toward reparative phenotypes (Shen et al., 2022). Meanwhile, excessive reactive oxygen species (ROS) accumulation promotes osteoclast-mediated bone resorption through NF-κB-related inflammatory pathways and impairs BMSC osteogenic commitment and angiogenic responses, further compromising bone regeneration under diabetic or inflammatory conditions (Liang et al., 2025). Consequently, the defect site may enter a self-perpetuating state of chronic inflammation, impaired osteogenesis and delayed repair (Sheng et al., 2023). Although the initiating mechanisms differ among pathological bone defects, persistent inflammatory signaling, defective inflammation resolution, impaired vascularized osteogenesis and disturbed bone remodeling represent common barriers to effective regeneration.

Taken together, bone regeneration is a temporally coordinated and spatially coupled process shaped by inflammation resolution, angiogenesis, osteogenic differentiation and bone remodeling. These intersystem interactions indicate that ideal bone-repair biomaterials should not merely provide passive structural support. Rather, they should actively modulate immune-regenerative timing and reshape the immune–vascular–osteogenic network to create a regenerative microenvironment (Xie et al., 2020; Zhao et al., 2022). Importantly, the dominant regulatory barriers and required intervention windows vary among critical-sized, infected, metabolic abnormality-associated, and oral and maxillofacial bone defects. This biological framework therefore provides the basis for designing functionalized GelMA-based hydrogels with stage-matched and pathology-adapted osteoimmunomodulatory functions, rather than using them simply as defect-filling scaffolds.

## 3 Osteoimmunomodulatory strategies of functionalized GelMA-based hydrogels

GelMA hydrogels provide a versatile and engineerable matrix for bone regeneration because they combine an extracellular matrix-like network, photocrosslinkable processability and the capacity for the local incorporation and delivery of bioactive components (Yue et al., 2015; Huang et al., 2025). However, pristine GelMA hydrogels are often insufficient as stand-alone matrices for complex bone repair, owing to their relatively weak mechanical strength, limited intrinsic osteoinductivity and inadequate ability to reshape pathological inflammatory microenvironments (Zhu et al., 2024d; Huang et al., 2025; Li G. et al., 2026). Therefore, the focus of GelMA-based bone-regenerative systems has shifted from passive cell support toward functionalized matrix design for active osteoimmunomodulation. Importantly, many of the functional agents discussed below are not intrinsically specific to GelMA. Their immunomodulatory, antioxidant, antibacterial or osteogenic activities should therefore be distinguished from the matrix-level contributions of GelMA, which include a photocrosslinkable, degradable and cell-interactive network that can support local retention, structural integration and controllable presentation of incorporated functional cues.

From this perspective, functionalized GelMA hydrogels should be viewed as immunoregenerative matrices rather than simple delivery carriers. Through the coordinated regulation of matrix properties and the rational incorporation of inorganic nanocomponents, bioactive molecules, extracellular vesicles, blood-derived products and microenvironment-responsive modules, GelMA matrices can be endowed with complementary immunomodulatory, angiogenic and osteogenic functions. The central design rationale is to resolve persistent inflammation and establish a microenvironment conducive to vascularized osteogenesis and subsequent bone remodeling, thereby supporting immune–vascular–osteogenic coupling during bone repair (Newman et al., 2021; Xu et al., 2024). In the following sections, we discuss major functionalization strategies for GelMA hydrogels, with particular emphasis on how matrix properties, incorporated functional components and responsive mechanisms influence immune regulation, vascularization, osteogenesis and bone-regenerative outcomes. The overall design framework is summarized in Figure 2.

**FIGURE 2:**
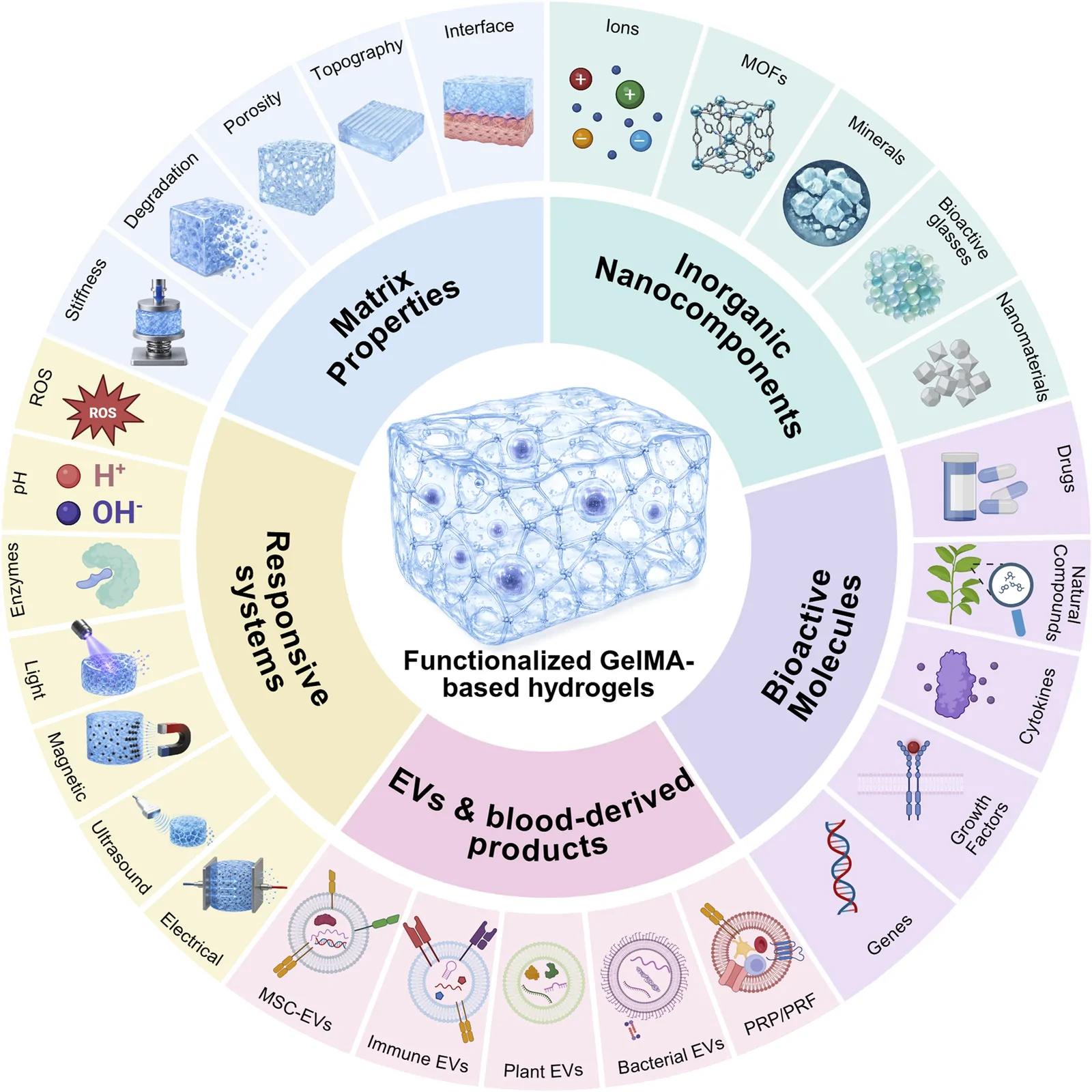
Osteoimmunomodulatory strategies of functionalized GelMA-based hydrogels. This schematic summarizes the major functionalization strategies by which functionalized GelMA-based hydrogels modulate the osteoimmune microenvironment and support bone regeneration, including matrix-property regulation; inorganic nanocomponents; bioactive molecules; extracellular vesicles and blood-derived products; and microenvironment-responsive systems. Created in BioRender. Chen, L. (2026) https://BioRender.com/rpgj7hr.

### 3.1 Matrix-mediated osteoimmunomodulation

The physicochemical properties of GelMA-based hydrogels represent the primary material-level interface through which implanted materials influence osteoimmune responses (Bupphathong et al., 2022; Yang T. et al., 2025). Before additional bioactive components are incorporated, matrix stiffness, degradation behavior, pore architecture, micro/nano-topography and interfacial stability determine how the material conforms to and maintains space within the defect, how immune and reparative cells infiltrate the scaffold, and how long local regulatory signals can be retained (Yang T. et al., 2025). Therefore, these properties should not be considered isolated material parameters, but as an integrated design space that links material mechanics, mass transport, cellular infiltration, matrix degradation and host–material interactions.

Matrix stiffness is a key biophysical cue for osteogenic commitment and immune regulation (Zhuang et al., 2020). By adjusting GelMA concentration, crosslinking density or reinforcing components, GelMA-based matrices with different mechanical properties can be constructed to regulate cell adhesion, cytoskeletal organization and osteogenic differentiation (Zhuang et al., 2020; Bupphathong et al., 2022). More directly, GelMA stiffness has been shown to regulate macrophage phenotype. In GelMA hydrogels spanning 2–29 kPa, increasing stiffness promoted greater macrophage spreading, F-actin and focal-adhesion organization, and a more pro-inflammatory M1-associated response *in vitro*; stiffer matrices were also associated with a stronger inflammatory response after implantation (Zhuang et al., 2020). These findings support stiffness as an immune-instructive mechanotransduction cue rather than simply a structural parameter. However, stiffness-related effects remain context-dependent because changes in crosslinking chemistry, porosity and matrix density may occur concurrently and influence macrophage responses (Yang T. et al., 2025). Importantly, stiffness should not be viewed only as an initial modulus but also as a time-dependent property that evolves during hydrogel degradation and tissue ingrowth. Zou et al. showed that a stiff GelMA matrix enhanced the osteogenic potential of human periodontal ligament cells (PDLSCs) and that this stiffness-induced phenotype could be partly retained after removal from the original mechanical environment, suggesting a mechanical memory effect during periodontal bone repair (Zou et al., 2026). This finding indicates that GelMA matrices should be designed to provide sufficient early mechanical instruction while avoiding excessively rapid softening before mineralized tissue formation (Zou et al., 2026). Thus, stiffness optimization should consider both the initial modulus and the time-dependent, degradation-associated loss of mechanical support.

Degradation rate is likewise a dynamic immunomodulatory cue because it continuously reshapes the physical microenvironment sensed by immune and reparative cells. In GelMA, protease-mediated network remodeling progressively changes matrix stiffness, pore accessibility, mass transport and the duration of cell–material interactions. If degradation proceeds too rapidly, mechanical and spatial cues may be lost before reparative tissue is sufficiently established; if it proceeds too slowly, cell infiltration and matrix replacement may be constrained (Huang et al., 2025; Yang T. et al., 2025). Accordingly, degradation kinetics should be aligned with the transition from inflammatory to reparative phases and treated as part of the immune-instructive matrix rather than merely as a measure of scaffold persistence.

Pore architecture and micro/nano-topography further regulate osteoimmune responses by controlling cell infiltration, nutrient diffusion and cell–cell communication (Yang T. et al., 2025). Highly interconnected pores facilitate the entry of immune cells, stem/progenitor cells and blood vessels, whereas poorly connected structures may restrict tissue ingrowth and maintain local inflammatory stress (Yang T. et al., 2025). However, pore design should not simply pursue larger pores or higher porosity, because excessive porosity may weaken mechanical integrity and defect-space maintenance (Bupphathong et al., 2022). A representative example is a bone-mimetic hydrogel with interconnected micropores and micro/nano-rough surfaces. Its porous structure promotes early cell infiltration, vascular ingrowth and cellular network formation, while the nanoscale roughness promoted macrophages toward a reparative M2 state through mechanotransduction-related RhoA/ROCK2 signaling (Song et al., 2024). This study suggests that pore interconnectivity and surface topography can actively shape the osteoimmune microenvironment rather than merely provide passive channels for tissue ingrowth.

Interfacial stability and conformal adaptation are also essential for GelMA-based osteoimmunomodulation, especially in irregular, particulate or mechanically dynamic defects. Unstable material–host contact, particulate micromotion or poor adaptation to the defect surface may prolong inflammatory irritation and impair bone regeneration (Wang et al., 2023). Photocrosslinkable GelMA has been used to stabilize Bio-Oss® particulates, improving particle aggregation, surgical handling and attachment to host bone defects. Compared with free particles, GelMA-stabilized particulates promoted macrophages toward an M2 phenotype, were associated with increased formation of mannose receptor-positive (MMR^+^) multinucleated giant cells, and enhanced vascular ingrowth and new bone formation (Wang et al., 2023). This observation indicates that reducing harmful micromotion and improving material–tissue contact can themselves contribute to a more favorable immunoregenerative environment.

Interface-oriented GelMA systems can also be designed as bioactive coatings or adhesive matrices. On titanium implants, a CMCSMA/GelMA hydrogel coating containing lithium silicate and selenium nanoparticles formed an adhesive layer that scavenged excessive ROS, promoted reparative macrophage polarization, improved antibacterial activity and enhanced osseointegration (Jiang et al., 2026). In this system, the conformal coating provided stable contact with the implant surface, while the incorporated inorganic components supplied additional redox-regulatory and antibacterial functions. For irregular bone defects, mussel-inspired adhesive GelMA-based hydrogels can conformally adhere to the defect surface and continuously release bioactive cues, thereby promoting a shift from inflammatory toward reparative macrophage states and improving bone integration (Liang et al., 2026). In periodontal defects, where masticatory loading, bacterial exposure and limited local retention coexist, an injectable double-network GelMA-based hydrogel reinforced with oxidized bacterial nanocellulose improved fatigue resistance and periodontal-pocket retention while enabling localized metronidazole delivery, reducing inflammatory infiltration and inhibiting alveolar bone resorption (Han Z. et al., 2025). This example highlights the importance of combining mechanical durability and local retention with bioactive delivery in mechanically dynamic and microbially exposed defects.

Collectively, the physicochemical regulation of GelMA-based hydrogels should be understood as an integrated material–cell–tissue design strategy. Matrix stiffness provides mechanical instruction to immune and osteogenic cells; degradation dynamically reshapes matrix mechanics, pore accessibility and the duration of cell–material interactions; pore architecture and micro/nano-topography organize cell infiltration and osteoimmune crosstalk; whereas interface engineering maintains local stability, improves defect adaptation and reduces chronic irritation. Future GelMA-based osteoimmunomodulatory hydrogels should therefore be optimized as dynamic matrices whose mechanical, degradative, structural and interfacial cues evolve in step with the requirements of bone repair, rather than as passive carriers for bioactive agents alone.

### 3.2 Inorganic nanocomponents

Inorganic nanocomponents are widely incorporated into functionalized GelMA hydrogels to compensate for the limited mechanical strength, mineralization capacity and microenvironmental adaptability of organic hydrogel networks alone. In the context of osteoimmunomodulation, their roles extend beyond structural reinforcement or passive mineral supplementation. Through ion release, mineralization induction, redox regulation, antibacterial activity and interfacial modulation, inorganic components can help coordinate inflammation resolution, vascularization and osteogenesis. According to their dominant functions, inorganic-component-functionalized GelMA hydrogels can be broadly divided into bioactive ions and mineralizing components, and multifunctional inorganic nanomaterials. To clarify the design logic of inorganic-component-functionalized GelMA hydrogels, Table 1 summarizes representative systems according to component category, functional component and immunoregenerative effects.

**TABLE 1:** Inorganic nanocomponents in functionalized GelMA hydrogels for osteoimmunomodulation and bone regeneration.

Category	Functional component	Immunoregenerative effects	Refs
Ions/MOFs	Zn^2+^/Ce^3+^co-doped whitlockite nanoparticles	Inhibit NF-κB signaling, suppress osteoclastogenesis and enhance osseointegration	(Jin et al., 2026)
	Sr-LDH + QK peptide	Suppress osteoclast activity, increase PDGF-BB expression and promote type H vessel formation	(He et al., 2025c)
	DFO@Zn-MOF-818	Restore mitochondrial homeostasis and activate HIF-1α/VEGF signaling to promote angiogenesis and osteogenesis	(Bai et al., 2025)
Minerals/BGs	CAT + BMP-2-loaded CaCO_3_ microspheres	Relieve hypoxia and inflammation while delivering BMP-2/Ca^2+^ to enhance osteogenesis	(Lu et al., 2024b)
	Sr-MBGNs	Suppress oxidative inflammation and coordinate M2 macrophage polarization, angiogenesis and osteogenesis	(Xu et al., 2023b)
	45S5 BG	Promote M2 macrophage polarization, angiogenesis and osteogenesis	(Ai et al., 2023)
	GOx-loaded oyster shell-derived nHAP	Promote M2 macrophage polarization, angiogenesis and osteogenesis	(Shen et al., 2025)
	ACZP	Suppress inflammatory macrophage responses and promote M2 macrophage response and bone regeneration	(Wang et al., 2025b)
	MBGNs	Inhibit MAPK/NF-κB signaling, reduce inflammation and accelerate bone repair	(Zhang et al., 2023)
Nanomaterials	SeNPs + Lap	Scavenge ROS, promote M2 macrophage polarization and improve osseointegration	(Jiang et al., 2026)
	ZnCe-LDO nanozyme + Yoda1	Suppress MAPK/TNF signaling and activate Piezo1/YAP1 signaling to enhance angiogenic osteogenesis	(Xiao et al., 2025b)
	BP nanosheets + MnCO nanosheets	Suppress pro-inflammatory macrophage responses and enhance M2 polarization and biomineralization	(Wu et al., 2026)
	L/D-cysteine-modified Fe_3_O_4_ nanoparticles	Regulate oxidative stress and macrophage polarization to support peri-implant bone regeneration	(Shi et al., 2026)
	Laponite + Apt19s	Recruit BMSCs, promote M2 macrophage polarization and activate AMPK/mTOR signaling to enhance osteogenesis	(Zhou et al., 2024)
	GO/Cu nanosheets + β-TCP	Promote M2 macrophage polarization and anti-inflammatory cytokine secretion, thereby enhancing angiogenesis and osteogenesis	(Yang et al., 2024)
	Lap@PDA nanosheets	Promote M2 macrophage polarization and activate BMP-2/TGF-β1 signaling to enhance angiogenesis and osteogenesis	(Wu et al., 2023)

#### 3.2.1 Bioactive ions and mineralizing components

Bioactive ions and metal–organic frameworks (MOFs) can provide local and sustained ion delivery within GelMA networks, thereby linking immunomodulation, antibacterial activity, angiogenesis and osteogenesis. ZIF-8 is one of the most representative MOF platforms because it can release Zn^2+^ and regulate the release of loaded bioactive components through its porous and responsive framework (Wang et al., 2020). ZIF-8-functionalized GelMA systems have been shown to reduce bacterial burden and inflammatory stimulation, promote reparative macrophage polarization, and enhance osteogenic differentiation of BMSCs or osteoprogenitor cells (Liu et al., 2022; Si et al., 2025b; Yu et al., 2025). For example, injectable ZIF-8/GelMA hydrogels sustained Zn^2+^ release, inhibited Porphyromonas gingivalis, reduced local inflammation and promoted alveolar bone regeneration in periodontitis-related defects (Liu et al., 2022). Beyond ZIF-8, other metal–organic frameworks have also been incorporated into GelMA systems, further broadening this strategy by coupling ion release with antioxidant, anti-inflammatory, angiogenic and osteogenic effects (He C. et al., 2025; Zhou Z. et al., 2025).

In addition to MOF-mediated delivery, the direct incorporation of bioactive ion-releasing components also provides important osteoimmune signals. Sr^2+^-related GelMA systems can regulate macrophage states, inhibit osteoclast activity and support H-type vessel-associated bone regeneration (He et al., 2025c; Xu et al., 2026). Mg^2+^ - functionalized systems can contribute to the regulation of inflammatory microenvironments by reducing ROS levels, suppressing inflammatory cascades and promoting M2 macrophage polarization, cell migration and angiogenesis (Sun et al., 2022; Pei et al., 2024). Other bioactive ion-releasing inorganic components, such as lithium-containing bioactive glasses and silicate-based materials, can further regulate macrophage behavior, angiogenesis and osteogenic differentiation under hyperglycemic, inflammatory or oxidative-stress microenvironments (Ma et al., 2022; Wu et al., 2022). Importantly, the effects of bioactive ions are not determined simply by increasing ion release. Rather than maximizing ion dosage, GelMA-based ion-releasing systems should aim for stage-matched and locally tolerable release to balance inflammation control, stem-cell recruitment, vascularization and bone formation, while avoiding excessive ion accumulation that may disturb microenvironmental homeostasis.

Bone-biomimetic minerals and bioactive glasses provide mineralizing components and osteoconductive signals for GelMA hydrogels. Hydroxyapatite and related mineral phases resemble the inorganic phase of natural bone and can enhance cell adhesion, osteogenic differentiation and mineral deposition while also contributing to local immune regulation (Cheng et al., 2024; Shen et al., 2025). Calcium-based mineral systems, including CaCO_3_ microspheres and amorphous calcium phosphate-based modules, can serve as Ca/P sources and carriers for functional components, thereby connecting early microenvironmental stabilization with later osteogenic mineralization (Lu K. et al., 2024; Wang S. et al., 2025). Bioactive glasses and mesoporous bioactive glass nanoparticles further integrate multi-ion release with bone-like interfacial bioactivity (Ai et al., 2023; Xu Z. et al., 2023). For instance, Sr-MBGNs/GelMA scaffolds combined Sr, Ca and Si ion release with the ECM-mimetic features of GelMA, thereby improving angiogenesis, osteogenesis, inflammatory regulation and matrix remodeling (Ai et al., 2023).

Overall, these studies suggest that bioactive ions and mineralizing components should be selected and combined according to their dominant functions, including immune regulation, vascular support, mineralization and mechanical reinforcement. Their incorporation should also be coordinated with GelMA degradation to meet the sequential requirements of bone repair.

#### 3.2.2 Multifunctional inorganic nanomaterials

As discussed above, pathological bone defects are often characterized by oxidative stress, infection-associated inflammatory stimulation, impaired vascularization and compromised osteogenic repair. Multifunctional inorganic nanomaterials are therefore introduced into GelMA systems to provide redox regulation, antibacterial activity and interfacial modulation within a single localized platform.

Redox-active inorganic nanomaterials and nanozymes can contribute to the restoration of osteoimmune homeostasis (Gao et al., 2024). Excessive ROS can amplify inflammatory signaling mediated by NF-κB, NLRP3 inflammasome activation and MAPK pathways, impair mitochondrial function and suppress osteogenic differentiation (Li et al., 2015; An et al., 2019). Inorganic nanomaterials with enzyme-mimetic activity can modulate ROS levels through variable valence states, catalytic surfaces or composite nanostructures, thereby reducing oxidative stress-driven inflammation and supporting macrophage and osteogenic cell function (Hui et al., 2024; Chen et al., 2025e; Shi et al., 2026). A representative example is the chiral Fe_3_O_4_/GelMA hydrogel. The D-type system enhanced Fe_3_O_4_ peroxidase-like activity and alleviated acute oxidative stress, whereas the L-type system promoted M2 macrophage polarization through the YTHDF1–m^6^A–Itgb3 axis, suggesting potential advantages under chronic immune–bone imbalance (Shi et al., 2026). This study suggests that chirality can serve as a stage-adaptive interfacial design factor rather than merely a compositional modification.

Antibacterial inorganic nanomaterials are particularly relevant for infected or microbiota-associated bone defects. By inhibiting bacterial adhesion, disrupting biofilm formation or reducing infection-induced inflammatory stimulation, these systems help shift the local microenvironment from persistent inflammation toward regeneration (Li J. et al., 2023; Wang L. et al., 2025). For example, arginine-derived carbon dots can be linked with GelMA hydrogels through Schiff base chemistry and released in infected microenvironments. They selectively kill bacteria while promoting a shift toward a reparative M2 macrophage state through IL-10 upregulation, thereby connecting antibacterial activity with immunomodulation and osteogenic repair (Li J. et al., 2023).

Inorganic nanomaterials can also act as functional interfaces within GelMA networks. Lamellar nanosheets, nanoclays and carbon-based nanomaterials can improve scaffold stability, rheological behavior and cell adhesion while regulating protein adsorption, cell recruitment and macrophage phenotype transition (Dutta et al., 2023; Wu et al., 2023; Yang et al., 2024). GelMA-CQDs, for instance, improve printability and near-infrared-responsive delivery, upregulate anti-inflammatory signals such as IL-4 and IL-10, promote M2 macrophage polarization, and support angiogenesis and osteogenic differentiation (Dutta et al., 2023). Similarly, two-dimensional nanosheets and nanoclays can be integrated into GelMA networks to combine mechanical reinforcement, interfacial regulation and bioactive delivery, thereby improving the coordination of inflammation control, vascularization and osteogenic repair (Li H. et al., 2024; Yang et al., 2024; Zhou et al., 2024).

Overall, the additional antibacterial, redox-regulatory, ion-mediated and osteogenic functions of these composite systems largely arise from the incorporated inorganic components, whereas GelMA provides a photocrosslinkable and cell-interactive matrix that can localize these components within the defect and integrate their delivery with scaffold structure and degradation. Depending on the formulation, the GelMA network can also contribute to local retention and release control. Functionalization therefore extends the matrix-level capabilities of GelMA rather than making these component-specific functions intrinsic properties of GelMA. Future designs should prioritize pathology-matched inorganic composition, controlled release and degradation–regeneration coupling to coordinate inflammation resolution, vascularization and bone formation.

### 3.3 Bioactive molecules

Bioactive molecules provide GelMA-based hydrogels with active regulatory capacity beyond matrix-mediated control. During bone repair, inflammation resolution, cell recruitment, angiogenesis and osteogenesis are coordinated by multiple biological signals (Rather et al., 2019). However, direct administration of these molecules is often limited by rapid diffusion, short local retention, instability and poor control of release kinetics. By using injectable, printable and degradable networks as local depots, GelMA hydrogels can improve molecular retention and enable more sustained or stage-matched regulation of the osteoimmune microenvironment (Li et al., 2024d; Yu C. et al., 2026). Because bioactive-molecule-loaded GelMA hydrogels regulate bone repair through diverse molecular cues, Table 2 compares representative systems according to molecule category, functional component and major immunoregenerative effects.

**TABLE 2:** Bioactive molecules in functionalized GelMA hydrogels for osteoimmune-mediated bone regeneration.

Category	Functional component	Immunoregenerative effects	Refs
drugs/natural compounds	AS	Suppress TLR4/TRAF6/IKK/NF-κB signaling and activate Wnt/β-catenin to promote osteogenesis	(Zhao et al., 2026)
	LDN-57444	Suppress UCHL1, restore mitochondrial–ER homeostasis and support osteogenesis	(Yuan et al., 2026)
	PP + DMOG	Suppress TNF/MAPK/NF-κB signaling, promote M2 polarization and enhance angiogenesis and osteogenesis	(He et al., 2026)
	G-Rb3	Rebalance MAPK and PI3K/Akt signaling to reduce inflammation and bone resorption	(Sun et al., 2025a)
	EGCG + CHX	Suppress IL-17/NF-κB signaling and limit inflammatory bone resorption	(Ge et al., 2025)
	ATRA-loaded ZIF-8	Limit M1 macrophage polarization, reduce inflammatory bone resorption and support osteogenesis	(Xiang et al., 2024)
cytokines/chemokines/growth factors	IL-10 + ICA	Couple early M2 polarization with sustained osteogenic differentiation	(Li et al., 2024d)
	SDF-1α+ IGF-1	Promote BMSC recruitment, M2 macrophage polarization and staged osteogenesis	(Zheng et al., 2025a)
	OSM	Promote M2 polarization, vascularization and osteogenesis	(Song et al., 2025c)
	SDF-1α+ OPG	Promote BMSC recruitment, M2 macrophage polarization and balanced bone remodeling	(Jiao et al., 2025)
	PGRN	Block TNF-α signaling, rebalance macrophage–T-cell crosstalk and promote bone repair	(Liu et al., 2024a)
	IL-4 + CGRP	Induce M2 macrophage polarization and enhance cAMP/PKA signaling for osteogenesis	(Wang et al., 2026a)
peptides/proteins/nucleic acids	ABL	Induce Foxp3^+^ Treg responses, enhance osteogenesis and limit osteoclastogenesis	(Jia et al., 2026)
	GCA-NAb	Block the GCA–Plxnb2–Arg2 axis, restore BMSC function and improve aged bone healing	(Dong et al., 2026)
	LK	Reduce inflammatory responses and promote vascularized bone regeneration	(Zhang et al., 2025b)
	SKP + OGP + KLT peptides	Recruit BMSCs and sequentially support angiogenesis and osteogenesis	(He et al., 2025d)
	pcDNA3.1-AKT	Activate AKT signaling, promote M2 macrophage polarization and support angiogenesis and osteogenesis	(Zhou et al., 2023)

#### 3.3.1 Drugs and natural compounds

Small-molecule drugs and natural compounds are attractive for GelMA functionalization because of their defined pharmacological activities, loading feasibility and dose adjustability. In inflammatory bone repair microenvironments, these molecules can reduce inflammation-mediated inhibition of osteogenesis and help restore reparative cell function. For example, artesunate-loaded GelMA hydrogels suppressed pro-inflammatory factor expression under LPS-induced inflammatory conditions and promoted BMSC osteogenic differentiation and mineralization (Zhao et al., 2026). Similarly, a GelMA hydrogel loaded with an inhibitor of ubiquitin carboxyl-terminal hydrolase L1 reduced macrophage-derived inflammatory cytokines, alleviated mitochondrial dysfunction and endoplasmic reticulum stress, and promoted alveolar bone regeneration (Yuan et al., 2026).

Natural bioactive compounds can further remodel the osteoimmune microenvironment through anti-inflammatory, antioxidant and macrophage-regulatory mechanisms. Oligomeric proanthocyanidins promoted a shift from an inflammatory macrophage phenotype toward a reparative M2 state and enhanced neovascularization and bone formation by reducing ROS levels (Zhang Y. et al., 2026). In periodontal inflammatory settings, GelMA@G-Rb3 alleviated LPS-induced inflammatory responses in PDLSCs through p38/ERK and PI3K/Akt-related signaling and reduced alveolar bone resorption (Sun J. et al., 2025). Under aging-related bone repair, GA@GelMA microspheres inhibited cellular senescence, promoted M2 macrophage polarization and enhanced BMSC osteogenic differentiation (Hong et al., 2025). These studies suggest that natural compounds in GelMA systems are not merely osteogenic adjuvants, but can act as microenvironmental regulators that connect inflammation control with osteogenic recovery.

Oxidative stress and metabolic dysregulation are also important targets for small-molecule functionalization. Deferoxamine-loaded GelMA systems promoted angiogenesis, reduced oxidative stress, induced M2 macrophage polarization and improved BMSC osteogenic function and osseointegration (Ai et al., 2024; Zhu et al., 2025). D-mannose-loaded GelMA hydrogels maintained BMSC osteogenic and chondrogenic potential by downregulating NLRP3 inflammasome activity and correcting inflammation-associated metabolic disturbance (Hu et al., 2026). More recently, GAPDH-targeted GelMA-based microspheres suppressed excessive glycolytic activation in macrophages and enhanced bacterial clearance in infected bone defects under diabetic conditions, indicating that immunometabolic reprogramming may extend GelMA-mediated regulation from phenotypic immunomodulation to mechanism-directed metabolic intervention (Jin et al., 2024).

Because bone repair is temporally organized, small-molecule delivery should also consider release sequence rather than sustained release alone. A hierarchical periosteum–bone scaffold containing dexamethasone and endothelial cell-derived components achieved staged regulation of angiogenesis, inflammation and mature bone formation (Yu C. et al., 2026). A biphasic scaffold loaded with quercetin and icariin exploited the different degradation rates of GelMA and PGCL to link early antioxidant immunomodulation with later osteogenic induction (Han C. et al., 2025). These examples indicate that the value of small-molecule delivery lies not only in selecting anti-inflammatory or osteogenic compounds, but also in matching their release windows with the changing requirements of bone repair.

#### 3.3.2 Protein, peptide and gene regulators

Compared with small molecules, proteins, peptides, cytokines, chemokines and nucleic acid regulators provide more direct control over intercellular communication, cell recruitment and gene expression (Szwed-Georgiou et al., 2023). Their direct administration is often limited by short half-life, inactivation and low delivery efficiency, whereas GelMA networks can improve local retention and provide a protected microenvironment for controlled delivery (Wang X. et al., 2025).

GelMA systems for chemokine and cytokine delivery are primarily designed to orchestrate endogenous reparative-cell recruitment and immune microenvironment remodeling. SDF-1α is commonly used to recruit endogenous stem/progenitor cells. When combined with osteoprotegerin, SDF-1α-loaded GelMA nanocomposite systems enhanced BMSC recruitment and osteogenic differentiation, promoted M2 macrophage polarization and helped maintain bone remodeling balance by inhibiting RANKL-related bone resorption (Jiao et al., 2025). Similarly, SDF-1α immobilized on core–shell microspheres and combined with spatiotemporal IGF-1 delivery enhanced BMSC chemotaxis, induced STAT6-associated M2 macrophage polarization and supported staged osteogenesis (Zheng J. et al., 2025). Anti-inflammatory cytokines can further accelerate the transition from inflammation to repair. IL-10-containing GelMA systems induced M2 macrophage polarization and enhanced BMSC migration and osteogenic capacity (Hu S. et al., 2024), whereas sequential IL-10 and icariin delivery coupled early inflammation resolution with later bone remodeling (Li et al., 2024d). IL-8 liposome-loaded GelMA scaffolds also promoted endogenous BMSC recruitment and M2 macrophage polarization (Wang X. et al., 2024).

Pleiotropic cytokines may be particularly useful when a single factor can simultaneously regulate recruitment, immune remodeling, angiogenesis and osteogenesis. Oncostatin M-loaded porous GelMA cryogel microspheres promoted BMSC migration and osteogenic differentiation, enhanced angiogenesis, induced M2 macrophage polarization, reduced osteoclast differentiation and promoted bone regeneration in diabetic calvarial defects (Song R. et al., 2025). This example suggests that cytokine delivery in GelMA systems should be evaluated not only by its anti-inflammatory efficacy, but also by its ability to coordinate immune–vascular–osteogenic coupling under pathological conditions.

Protein and peptide regulators can also correct abnormal osteoimmune states or provide targeted bioactive signals. Local delivery of a grancalcin-neutralizing antibody by GelMA hydrogel alleviated aged macrophage-mediated BMSC dysfunction and improved age-related bone healing (Dong et al., 2026). Functional peptides such as stem-cell-homing peptide, angiogenic peptide and osteogenic growth peptide have been incorporated into bone marrow-mimetic systems to promote cell recruitment, vascular maturation and bone regeneration (He Z. et al., 2025). Lumbrokinase-loaded GelMA hydrogels enhanced angiogenesis and osteogenesis while reducing local inflammatory responses (Zhang Y.-W. et al., 2025). In infection-related microenvironments, antimicrobial peptide-grafted GelMA microspheres reduced bacterial burden and, together with hypoxia-related signals, promoted stem cell osteogenic differentiation and tissue repair (Chen et al., 2025d).

Nucleic acid delivery and engineered-cell strategies further expand GelMA-based regulation from extracellular signal delivery to gene-level intervention. circAars-engineered ADSCs loaded onto GelMA scaffolds enhanced osteogenesis through miR-128-3p-related regulation and promoted M2 macrophage polarization and angiogenesis through paracrine mechanisms (He et al., 2025b). IL-4 mRNA-engineered monocytes combined with icariin-loaded GelMA microspheres enabled early local IL-4 production for immune reprogramming, followed by sustained icariin release to inhibit multinuclear fusion and osteoclast-related processes (Wu et al., 2025). Other gene-regulatory approaches, including Sox9-targeted siRNA, AKT-related gene delivery and CpG nucleic acid analogs, further provide routes for regulating macrophage polarization, osteoclast activity and osteogenic–angiogenic coupling (Zhou et al., 2023; Rahmani et al., 2025; Tan et al., 2026).

Taken together, bioactive molecules expand the regulatory scope of GelMA-based hydrogels by introducing pharmacological, cytokine-mediated and gene-level cues into local bone repair. In these systems, the primary pharmacological, cytokine or gene-regulatory activities arise from the delivered molecules, whereas GelMA can reduce rapid local diffusion, improve retention, provide structural integration and couple delivery with matrix degradation, while remaining compatible with injectable or printable fabrication. Their value, however, should not be judged simply by the number of incorporated signals. More important is whether the selected cues match the dominant pathological barrier, whether their release sequence follows the transition from inflammation to regeneration, and whether their bioactivity can be maintained without disturbing the balance among immune remodeling, vascularization and osteogenesis.

### 3.4 Extracellular vesicles and blood-derived products

Extracellular vesicles (EVs) and blood-derived products are endogenous sources of bioactive signals that can be incorporated into GelMA hydrogels to regulate the osteoimmune microenvironment. Compared with single recombinant factors or synthetic molecules, these biologically derived components contain multiple proteins, nucleic acids, cytokines and growth factors, thereby enabling broader regulation of inflammation, angiogenesis, cell recruitment and osteogenesis (Biswas et al., 2025; Mormone et al., 2026). However, their direct application is often limited by poor local retention, uncontrolled release and loss of bioactivity. GelMA hydrogels can partially address these limitations by providing spatial confinement, microenvironmental protection and sustained release (Man et al., 2022).

#### 3.4.1 Extracellular vesicles

EVs mediate intercellular communication through the transfer of proteins, lipids and nucleic acids, and have therefore been widely explored as cell-free regulators of bone repair. In GelMA-based systems, EVs can be locally retained and gradually released, thereby prolonging their effects on immune, endothelial and osteogenic cells. Given the increasing use of EVs as cell-free immunoregulatory cues, Table 3 summarizes representative mammalian cell-derived EVs incorporated into GelMA-based systems according to vesicle source, functional component and immunoregenerative effects.

**TABLE 3:** Extracellular vesicles in functionalized GelMA hydrogels for osteoimmune-mediated bone regeneration.

Source	Functional component	Immunoregenerative effects	Refs
MSCs	MSC-Exos + ZIF-8	Promote osteogenesis and suppress NF-κB-dependent inflammation through the miR-23a-3p/PTEN/AKT axis	(Si et al., 2025b)
	MSC-Exos + Curcumin	Enhance DNA repair, reduce oxidative injury and promote M2 macrophage polarization	(Si et al., 2025a)
	MSC-EVs	Regulate macrophage polarization and promote angiogenesis and osteogenesis	(Li et al., 2025a)
M2 macrophage	M2-EVs + BMP-2	Promote M2 macrophage polarization and BMP-2-enhanced vascularized osteogenesis	(Ling et al., 2025)
	M2-EVs	Suppress NEK7/NLRP3 inflammasome activation and reduce macrophage pyroptosis	(Cheng et al., 2026)
	M2-exos + Melatonin	Suppress ER stress and chronic inflammation while restoring osteogenic potential	(Cui et al., 2023)
Neutrophil	MP196 + Neutrophil-derived exosomes	Promote neutrophil apoptosis, efferocytosis and M2 macrophage polarization	(Zhuo et al., 2025)
USCs	USC-Exos + DBM	Activate AMPK signaling and promote M2 macrophage polarization, angiogenesis and osteogenesis	(Chen et al., 2026)
hDPSCs	Tet213 + hDPSCs-Exos	Coordinate M2 macrophage polarization, antibacterial activity and osteogenic signaling	(Zhang et al., 2026a)
HUVECs	HUVECs-Exos	Inhibit NLRP3 inflammasome activation and promote vascularized bone regeneration	(Chen et al., 2023)

Mammalian cell-derived EVs remain the most commonly used EV source in GelMA-based bone repair systems. MSC-derived EVs are particularly representative because they can promote osteogenesis while modulating macrophage polarization. After donor-cell preconditioning or engineering, their ability to regulate M2 macrophage polarization, angiogenesis and osteogenic signaling can be further enhanced (He et al., 2024; Li et al., 2024c; Li L. et al., 2026). For example, osteogenically preconditioned MSC-derived exosomes incorporated into GelMA hydrogels supported sustained local delivery, enhanced BMSC osteogenic differentiation and endothelial angiogenesis, and promoted M2 macrophage polarization under inflammatory conditions (Li et al., 2024c). Curcumin-engineered MSC exosomes further attenuated ROS-related damage, inhibited osteoclastic activity and promoted M2 macrophage polarization (Si et al., 2025a). Other mammalian EVs, including those derived from hypoxia-preconditioned cartilage progenitor cells, NEAT1-enriched endothelial cells and urine-derived stem cells, can also support immune homeostasis and tissue regeneration by regulating autophagy, inflammasome activation or energy metabolism (Chen et al., 2023; 2026; Zhou P. et al., 2026).

Immune cell-derived EVs provide a more direct route for osteoimmunomodulation. M2 macrophage-derived EVs can suppress inflammasome activation through the miR-23a-3p/NEK7/NLRP3/IL-1β axis and promote BMSC osteogenic differentiation (Cheng et al., 2026). Melatonin-engineered M2 macrophage-derived EVs further alleviate endoplasmic reticulum stress and restore the osteogenic and cementogenic differentiation capacity of hPDLCs under inflammatory conditions (Cui et al., 2023). These studies indicate that immune-cell-derived EVs can transmit the immunoregulatory characteristics of their parent cells and reshape the local inflammatory niche.

Plant-derived EVs and engineered bacterial EVs further broaden the source spectrum of GelMA-based EV systems. Lemon-derived nanoparticle-functionalized GelMA/DAS hydrogels promoted M2 macrophage polarization, suppressed NF-κB-related inflammatory signaling, and improved collagen remodeling and angiogenesis (Jin et al., 2025). Drynaria rhizome-derived EVs combined with sinomenine and GelMA-containing microspheres enhanced osteogenesis, inhibited osteoclastogenesis, and promoted M2 macrophage polarization and Treg-associated immunoregulation (Cai L. et al., 2026). More recently, bioengineered bacterial EVs carrying pBMP-2-VEGF plasmids were co-encapsulated with IL-4 in GelMA hydrogels. Early IL-4 release promoted M2 macrophage polarization and alleviated chronic inflammation, whereas subsequent BEV-mediated BMP-2/VEGF expression enhanced osteogenesis and vascularization, accelerating osteoporotic fracture repair (Zhou G. et al., 2025). Together, these studies suggest that EV-loaded GelMA systems can evolve from passive EV depots into programmable platforms for coordinated immune, vascular and osteogenic regulation.

#### 3.4.2 Blood-derived products

Blood-derived products, including platelet-rich plasma (PRP), platelet-rich fibrin (PRF), injectable platelet-rich fibrin (i-PRF) and autologous platelet concentrates (APCs), provide complex mixtures of endogenous growth factors, cytokines and matrix-associated proteins. Their advantages include the potential for autologous use, good biocompatibility and low immunogenicity (Ding et al., 2021). However, their biological activity is often transient, and their release behavior is difficult to control. GelMA hydrogels can improve their structural stability and prolong their local effects during bone repair.

PRP-GelMA hydrogels have been used to link platelet-derived biological activity with hydrogel-mediated structural support. A 20% PRP-GelMA hydrogel promoted M2 macrophage polarization, increased repair-associated markers such as Arg1 and CD206, and enhanced BMSC proliferation, migration and osteogenic differentiation (Jiang et al., 2021). In 3D printing systems, PRP or i-PRF can also be integrated into GelMA/AlgMA matrices and further modified with laponite. This strategy delayed the release of multiple platelet-derived growth factors, while laponite-derived bioactive ions synergized with endogenous growth factors to promote M2 macrophage polarization, BMSC recruitment, vascularization and osteogenic differentiation (Cao et al., 2023; 2025). In i-PRF-AGM@Lap systems, the osteogenic effects on BMSCs were further associated with miR-21- and miR-125a-mediated TGF-β/Smads signaling (Cao et al., 2025).

PRF/i-PRF-based GelMA systems can also stabilize the local repair microenvironment. Hep/GelMA-PRF regulated growth factor release, improved degradation behavior, alleviated local inflammation and upregulated osteogenesis-related proteins (Tao et al., 2024). Beyond growth factor delivery, APC-based CurCDs@iPRF-MA remodeled macrophage metabolism under inflammatory conditions by regulating mitochondrial homeostasis and oxidative phosphorylation, while also scavenging ROS and promoting angiogenesis and tissue repair (Ren et al., 2025). These findings suggest that blood-derived products in GelMA systems should not be viewed only as growth factor reservoirs, but also as biologically complex regulators of inflammation, vascularization and osteogenic signaling.

Taken together, EVs and blood-derived products introduce biologically derived multicomponent signals into GelMA systems, but their regenerative value is not determined simply by biological origin or cargo complexity. Their intrinsic immunoregulatory and regenerative activities are not specific to GelMA; rather, the GelMA matrix can provide spatial confinement, improve local retention, help protect these biological cargos within the local microenvironment and enable their integration into injectable or printable constructs. More important is whether GelMA-based delivery can help preserve bioactivity, regulate release kinetics and align functional outputs with the temporal sequence of inflammation resolution, vascularization and osteogenesis. For future translation, source standardization, batch-to-batch consistency and release–function relationships should be addressed as key design and quality-control criteria.

### 3.5 Microenvironment-responsive GelMA systems

During bone repair, immune status, tissue metabolism and regenerative demands change dynamically with inflammation resolution, vascular ingrowth and bone reconstruction (Loi et al., 2016). Microenvironment-responsive GelMA systems are designed to couple cargo release, material degradation or functional activation with these local changes, thereby improving the spatiotemporal precision of osteoimmunomodulation. According to the source of triggering signals, these systems can be broadly divided into endogenous pathological triggers and exogenous physical stimuli, whereas multi-stimuli-responsive systems integrate both types of triggers to achieve more complex staged regulation.

Importantly, spatiotemporal immune control should not be equated with continuous suppression of inflammation. Rather, GelMA-based systems should preserve the transient inflammatory activity required for early repair while facilitating timely resolution and subsequent reparative signaling through sequential release, degradation-dependent exposure or stimulus-gated intervention. Given the continuum of macrophage activation states, these strategies are more appropriately interpreted as stage-matched regulation of inflammatory-to-reparative programs rather than as a rigid M1-to-M2 switch (Loi et al., 2016; Li et al., 2024d; Wang L. et al., 2025; Yu Y. et al., 2026).

#### 3.5.1 Endogenous pathological triggers

Pathological bone-defect microenvironments can provide endogenous triggers, including elevated ROS, local acidity and increased enzymatic activity, for the design of responsive GelMA-based systems (Wei et al., 2022). These signals reflect inflammatory or metabolic disturbances, but they can also be used as pathology-derived inputs for on-demand release and functional activation in GelMA-based systems.

ROS-responsive systems are mainly used to relieve oxidative stress and reshape the inflammatory microenvironment. For example, GGBH@EAC hydrogels use boronate ester bond cleavage under high-ROS conditions to release EGCG-Arg-Cu nanoparticles. The released components inhibit BMSC ferroptosis by restoring redox homeostasis and reprogramming lipid metabolism, thereby enhancing osteogenic differentiation and vascularized bone regeneration (Li C. et al., 2026). Other ROS-responsive GelMA systems release bioactive molecules such as METRNL, CGRP or STING inhibitors to reduce ROS levels, regulate macrophage polarization and improve angiogenic and osteogenic responses (Sun Z. et al., 2025; Xu Y. et al., 2025; Cai Z. et al., 2026). In aging-related inflammatory settings, the FMAG nanoplatform combines ROS-responsive fisetin release with MnO_2_ nanozyme-mediated ROS scavenging, promoting senescent cell clearance and macrophage efferocytosis through the MerTK/Gas6 axis (Wang Z. et al., 2025). These examples suggest that ROS-responsive design should not be viewed only as an antioxidant intervention, but as a way to reconnect redox regulation with immune remodeling and osteogenesis.

Acidic pH provides another endogenous trigger, especially in inflammatory, infectious and bone resorption-related microenvironments. Biomimetic hematoma microspheres with a pH-responsive GelMA outer layer achieve spatiotemporally controlled H_2_S release under acidic conditions, thereby reducing chronic inflammation, promoting angiogenesis, activating autophagy and enhancing BMSC osteogenic differentiation (Zhou C. et al., 2026). Acid-responsive GelMA systems can also enhance endogenous MSC recruitment and angiogenesis by releasing SDF-1α and promoting NO generation or inhibit osteoclast activity through pH-sensitive naringin release (Gong S. et al., 2024; Xiao et al., 2025a). In infected repair environments, acid-triggered systems can release antibacterial components while promoting macrophage transition toward a reparative M2-like state, supporting subsequent osteogenic repair (Li J. et al., 2023; Qi et al., 2026).

Enzyme-responsive systems exploit elevated MMP or collagenase activity in pathological tissues to achieve localized release. MMP-2-responsive GelMA-IL-4 coatings release IL-4 in inflammatory microenvironments and synergize with Mg^2+^ to regulate macrophage metabolism, promote M2 macrophage polarization, reduce local immune responses and enhance vascularized bone regeneration (Bi et al., 2025). Collagenase-responsive D-lip/CM@GM microspheres degrade under collagenase stimulation and release DMF liposomes. By inhibiting glycolysis-related GAPDH in both macrophages and bacteria, this system links antibacterial activity with the regulation of inflammatory metabolism (Jin et al., 2024). MMP-responsive microhydrogels have also been used to release IL-4, SDF-1 or mRNA-based components, enabling immunomodulation, cell recruitment and regulation of osteogenic–osteoclastic homeostasis (Zheng et al., 2024; Chen B. et al., 2025).

Overall, endogenous ROS, acidic pH and enzyme activity allow GelMA-based systems to translate pathological cues into localized therapeutic responses. However, future designs should define trigger thresholds more clearly and avoid assuming that stronger responsiveness necessarily leads to better regeneration.

#### 3.5.2 Exogenous physical stimuli

Compared with endogenous triggers, exogenous physical stimuli can be controlled externally with respect to timing, intensity and spatial range. In GelMA-based systems, light, magnetic fields, ultrasound and electrical or piezoelectric cues are used to regulate antibacterial activity, cargo release, immune remodeling, angiogenesis and osteogenesis.

Near-infrared (NIR)-responsive systems are among the most widely used physical stimulus-responsive strategies. They usually rely on photothermal or photosensitive components such as polydopamine, black phosphorus, MXene or carbon quantum dots. LF-modified polydopamine-coated Ti_3_C_2_ MXene nanosheets incorporated into GelMA/PEGDA hydrogels released LF under 808 nm irradiation and activated HIF-1α/VEGF and PI3K/Akt-related signaling, thereby enhancing ROS scavenging and regulating immune responses, angiogenesis and osteogenesis (Liao et al., 2026). BP@PDA-Pd/GelMA systems further enhanced enzyme-like antioxidant activity under mild photothermal stimulation, scavenged excessive ROS and promoted M2 macrophage polarization (Song et al., 2025b). Similarly, GelMA scaffolds containing polyphenol carbon quantum dots enable NIR-triggered drug release while promoting M2 macrophage polarization and vascularized osteogenesis (Dutta et al., 2023).

Magnetically responsive GelMA systems mainly use magnetic nanoplatforms to generate magnetothermal effects or mechanically controlled deformation. Cascade magnetic hyperthermia systems can first use a high-temperature antibacterial stage to disrupt biofilms and then shift to mild magnetic hyperthermia to promote osteogenesis and suppress excessive inflammation (Wang L. et al., 2025). This staged temperature strategy is important because infection control and tissue regeneration require different thermal windows. Beyond magnetothermal therapy, magnetically responsive GelMA artificial periosteum can undergo tensile deformation under an external magnetic field, mimicking periosteal mechanical cues and promoting cell alignment, migration, angiogenesis, osteogenic differentiation and macrophage phenotype regulation (Dong et al., 2025).

Ultrasound-responsive systems offer deeper penetration and noninvasive spatiotemporal control. LNT-SeNPs@Gel uses ultrasound to promote selenium nanoparticle release during the inflammatory stage, reducing excessive ROS and inflammatory factor production, while later enhancing osteogenesis through selenoprotein-related PI3K/Akt signaling (Chen et al., 2025f). A coaxially printed hydrogel scaffold uses ultrasound to cleave thioketal linkages for controlled IL-10 release, while the core layer continuously releases Mg^2+^, thereby connecting early immunomodulation with later angiogenic and osteogenic induction (Yu Y. et al., 2026). In sonodynamic antibacterial systems, sonosensitizers combined with GelMA can enhance antibacterial ROS generation under ultrasound and, together with metformin and BMP-2, reduce inflammatory responses and bone loss (Hu B. et al., 2024).

Electrical and piezoelectric systems aim to mimic the electrophysiological and electromechanical features of bone tissue. Icariin-loaded GelMA hydrogels combined with potassium sodium niobate/nano-hydroxyapatite/poly (lactic acid) piezoelectric scaffolds generate stable piezoelectric output under ultrasound activation, promoting BMSC osteogenesis, endothelial migration and M2 macrophage polarization (Wang Y. et al., 2025). Injectable PiezoGEL integrates GelMA with barium titanate fillers and generates electrical cues under biomechanical stimulation such as mastication or tissue movement, while exerting antibacterial and osteogenic effects (Roldan et al., 2023). Recently, a GF-OsG smart bio-battery converted pathological high glucose into local microcurrents, inducing M2 macrophage reprogramming and enhancing BMSC osteogenic differentiation in diabetic bone defects (Lv et al., 2026). This strategy illustrates how pathological metabolic signals can be transformed into therapeutic electrical stimulation, although long-term electrical stability and biosafety remain to be clarified.

Multi-stimuli-responsive GelMA systems combine endogenous pathological triggers with exogenous physical stimuli to achieve staged regulation. NIR/magnetic dual-responsive 3D-printed scaffolds use photothermal effects for infection control and magnetic stimulation for angiogenesis–osteogenesis coupling (Zeng et al., 2025). NIR/pH-responsive photothermal hydrogels release DFO and phosphate ions while scavenging ROS, inducing M2 macrophage polarization and promoting vascularized osteogenesis (Wu et al., 2024). Other systems integrate photothermal, antibacterial, antioxidant or nanozyme functions to coordinate infection control, inflammation alleviation and bone regeneration (Li Q. et al., 2024; Liu et al., 2025a; Xiu et al., 2025). Although these designs improve adaptability to complex defects, they also make mechanistic attribution and stimulation-parameter optimization more difficult.

Responsive GelMA systems should be optimized within a defined therapeutic window rather than by maximizing stimulus intensity. For photothermal or magnetothermal strategies, antibacterial efficacy should be balanced against thermal injury to surrounding immune, endothelial and osteogenic cells, whereas pH-, ROS- or enzyme-responsive systems require activation thresholds that discriminate pathological from physiological conditions and minimize off-target release. Because these safety margins depend on formulation, stimulation duration and anatomical site, they should be defined in an indication-specific manner (Wei et al., 2022; Wang L. et al., 2025; Zeng et al., 2025).

Taken together, microenvironment-responsive GelMA systems provide a means to match therapeutic activation with the evolving repair microenvironment. In many of these systems, particularly those responding to ROS, light, magnetic fields, ultrasound or other engineered stimuli, the sensing or stimulus-responsive function is introduced by additional responsive components or chemical modifications rather than by pristine GelMA alone. In contrast, the protease-sensitive nature of the GelMA network itself can contribute to enzyme-dependent degradation and remodeling. Thus, the contribution of GelMA should be considered in a formulation-specific manner: it provides a photocrosslinkable, cell-interactive and protease-degradable matrix into which responsive elements can be integrated, while the actual trigger-sensing mechanism may derive from either the chemically modified GelMA network or the incorporated functional component (Yue et al., 2015; Wei et al., 2022). Future development should focus less on accumulating multiple triggers and more on defining response thresholds, stimulation windows, safety boundaries and the hierarchy and interactions among different responsive mechanisms.

## 4 Applications across distinct bone defect models

Bone defects arising under different pathological conditions differ in their dominant regenerative barriers, ranging from insufficient endogenous repair in critical-sized defects to infection-driven inflammation, metabolic dysregulation and oral/maxillofacial interface complexity. These differences require pathology-adapted design of functionalized GelMA hydrogels to achieve osteoimmunomodulation and bone regeneration. Accordingly, this section discusses their applications in four major scenarios: critical-sized, infected, metabolic disorder- and aging-associated, and oral/maxillofacial bone defects.

To make this comparison mechanistically explicit, the following sections evaluate each defect class according to four interrelated considerations: defect size and the associated requirements for mechanical support and space maintenance; anatomical location and the host–material interface; the dominant pathological microenvironment; and the stage of repair at which intervention is required. Accordingly, the same functional component may have different design value across defect contexts. This framework is intended to clarify how GelMA matrix properties and incorporated functions should be matched to the dominant regenerative barrier, rather than to imply a single universal “defect-adapted” solution.

### 4.1 Critical-sized bone defects

Critical-sized bone defects exceed the intrinsic self-healing capacity of bone and usually require biomaterials that provide structural support, local biological signals and osteoimmune microenvironment modulation (Su et al., 2022). In this context, functionalized GelMA hydrogels are not only used as defect fillers, but also as adaptable platforms for coordinating inflammation resolution, vascular ingrowth and osteogenesis.

Structural biomimicry is a major design route for critical-sized defect regeneration. A hierarchical periosteum–bone scaffold integrating a GelMA/BG/DEX hydrogel body with a periosteum-like layer promoted revascularization, anti-inflammatory regulation and mature bone formation in a rat calvarial defect model (Yu C. et al., 2026). A gradient 3D-printed BMGP scaffold further combined cancellous bone–cortical bone-like architecture with BP/MnCO nanosheet-loaded GelMA hydrogel, in which MnCO mediated anti-inflammatory effects and M2 macrophage polarization, while BP degradation promoted *in situ* mineralization (Wu et al., 2026). For irregular defects with unstable interfaces, mussel-inspired adhesive GelMA double-network hydrogels improved defect conformity and sustained the release of silicon ions and alendronate, thereby inducing M2 macrophage polarization and enhancing bone integration (Liang et al., 2026). These studies suggest that structural design in critical defects should be integrated with immune regulation and mineralization, rather than serving as mechanical support alone.

Local delivery of regenerative and immunomodulatory signals is another important strategy. MSC-Exos/ZIF-8@GelMA systems enhanced osteogenesis and angiogenesis while maintaining a relatively low inflammatory state in rat calvarial defects (Si et al., 2025b). PRP-GelMA/AlgMA systems modified with laponite prolonged the release of platelet-derived growth factors and promoted vascular ingrowth and bone regeneration (Cao et al., 2023). Core–shell microspheres with surface-immobilized SDF-1α and sustained IGF-1 release enhanced BMSC chemotaxis, reduced inflammation and supported staged osteogenesis (Zheng J. et al., 2025). These examples show that GelMA-based delivery can partially compensate for insufficient endogenous signals by linking stem cell recruitment, vascularization and immune remodeling.

Because inflammation is required to initiate repair but becomes detrimental when persistent, staged osteoimmunomodulation is particularly relevant for large defects. LPS/GelMA combined with OPC/PLA first recruited and activated macrophages and then promoted M2 macrophage polarization, thereby enhancing osteogenesis-related factor expression (Zhang Y. et al., 2026). Sequential release strategies, such as early immunomodulation followed by osteogenic or angiogenic stimulation, further approximate the temporal pattern of natural bone healing (Luo et al., 2025; Yu C. et al., 2026). Therefore, in critical-sized defects, the key value of functionalized GelMA hydrogels lies in integrating structural adaptation, signal delivery and immune-stage regulation into one coordinated regenerative process.

Overall, critical-sized defects are primarily constrained by insufficient endogenous repair, prolonged requirements for defect-space maintenance and the need to establish vascularized tissue across a large regenerative gap. Accordingly, the most relevant matrix-level advantages of GelMA in this setting are conformal defect filling, compatibility with injectable or printable fabrication, local retention of regenerative cues and integration with mechanically reinforcing phases, whereas incorporated functional components provide additional immunomodulatory, angiogenic or osteogenic signals. However, pristine GelMA remains insufficient for sustained load-bearing support, and its degradation must be matched to the time required for tissue infiltration, mineralized matrix formation and maintenance of defect space. Current evidence is still dominated by small-animal calvarial, femoral and femoral-condyle models; future studies should therefore emphasize long-term remodeling, load-bearing validation, degradation–regeneration matching, large-animal studies and clinically relevant handling properties.

### 4.2 Infected bone defects

Infected bone defects are characterized by bacterial persistence, biofilm formation and chronic inflammatory stimulation, all of which impair vascularization and osteogenesis. Therefore, functionalized GelMA hydrogels for infected defects should not only eliminate pathogens, but also guide the local osteoimmune microenvironment from infection-driven inflammation toward bone regeneration.

Early infection control remains the first prerequisite. GDZH hydrogels incorporating DHA@ZIF-8/HA nanoparticles into GelMA synchronously release DHA and Zn^2+^ in infected acidic microenvironments. DHA reduces bacterial burden and inflammation, while Zn^2+^ supports BMSC osteogenic differentiation, enabling repair of *Brucella*-infected bone defects (Qi et al., 2026). Bacterial enzyme-responsive CaP@MS-Oligo-Van microspheres further provide infection-triggered antibiotic release and subsequent CaP-supported bone repair (Lu G. et al., 2024). These examples indicate that antibacterial delivery should be coupled with inflammation alleviation and osteogenic support.

For infected defects with biofilms, oxidative stress or acidic microenvironments, responsive GelMA systems can organize treatment in a more stage-specific manner. NIR/magnetic dual-responsive scaffolds first eliminate MRSA through photothermal effects and then use ROS scavenging, inhibition of M1 macrophage polarization and magnetic stimulation to support angiogenesis–osteogenesis coupling (Zeng et al., 2025). Cascade magnetic hyperthermia systems similarly use an initial high-temperature antibacterial stage to disrupt biofilms, followed by mild magnetic hyperthermia and siCkip-1 release to promote osteogenesis and suppress excessive inflammation (Wang L. et al., 2025). In chronic osteomyelitis, GC-dECM@CPN cryogels combine NIR irradiation with acidic infection-responsive regulation; after bacterial clearance, the decellularized extracellular matrix further promotes BMSC osteogenic differentiation and M2 macrophage reprogramming (Liu et al., 2025a). These studies support a shift from simple antibacterial therapy toward sequential infection control, immune reprogramming and bone regeneration.

After infection clearance, tissue regeneration still depends on vascularization, osteogenic differentiation and remodeling capacity. GCS nanocomposite hydrogels use temporally regulated SQD and CPO release to integrate bacterial inactivation, ROS scavenging, M2 macrophage polarization, vascular coupling and osteogenic differentiation (Liu J. et al., 2025). BHM@CG hydrogels further combine adhesive hemostasis, antibacterial activity, ROS scavenging and sustained release of BMP-2-derived peptides to support infected critical-sized defect repair (Chen J. et al., 2025). Thus, functionalized GelMA hydrogels for infected bone defects should be evaluated not only by antibacterial efficiency but also by their ability to restore immune homeostasis and sustain downstream regeneration.

Overall, infected bone defects are distinguished by the need to control persistent pathogens and biofilms before a stable regenerative microenvironment can be established. Their design priority is therefore sequential rather than simply multifunctional: an early antibacterial phase must sufficiently reduce microbial burden and infection-driven inflammatory stimulation, followed by a cytocompatible phase that supports inflammation resolution, vascular recovery and osteogenic repair. In this context, GelMA is particularly useful as a localizable and conformal matrix for integrating antibacterial, responsive and regenerative modules, but the intensity and duration of antibacterial treatment must be balanced against potential damage to host immune, endothelial and osteogenic cells. Future studies should therefore define how antibacterial efficacy, immunomodulatory timing, material degradation and bone regeneration are matched under different infection states, while also evaluating infection recurrence, long-term safety and efficacy in clinically relevant large-animal models.

### 4.3 Metabolic abnormality-associated bone defects

Bone defects associated with metabolic disorders or aging are not merely local injuries occurring in systemically compromised hosts. Their regeneration is constrained by systemic and local pathological microenvironments, including hyperglycemia, oxidative stress, chronic inflammation, impaired angiogenesis, excessive osteoclastogenesis and cellular senescence (Pignolo et al., 2021; Iantomasi et al., 2023; Sheng et al., 2023). Therefore, functionalized GelMA hydrogels in these models should focus on osteoimmune microenvironment correction rather than osteogenic stimulation alone.

In diabetic bone defects, hyperglycemia, ROS accumulation and chronic inflammation reinforce one another, resulting in dysregulated M1/M2 macrophage polarization, mitochondrial dysfunction, insufficient vascularization and reduced osteogenic potential (Shen et al., 2022; Sheng et al., 2023). CS-Li/GelMA hydrogels promote macrophage transition toward an anti-inflammatory phenotype and improve angiogenesis and osteogenesis under high-glucose conditions, possibly through GSK-3β/β-catenin-related regulation (Xu C. et al., 2025). ROS- responsive and pH-responsive GelMA systems incorporating Cu^2+^, MgH_2_, Ce-based nanomaterials, CGRP or NO-generating modules can further relieve oxidative stress, regulate macrophage phenotype, restore mitochondrial homeostasis and enhance vascularized bone formation (Pei et al., 2024; Zhu et al., 2024c; Shi et al., 2025; Zhou Z. et al., 2025; Cai Z. et al., 2026). Glucose-responsive or glucose-consuming systems, such as Au@Pt nanozyme-loaded GelMA microspheres and GOx-containing scaffolds, directly target hyperglycemic stress while scavenging ROS and protecting BMSC osteogenic and paracrine functions (Gong Y. et al., 2024; Hui et al., 2024; Liu Z. et al., 2024; Feng et al., 2025). In addition, OSM-functionalized GelMA cryogel microspheres promote BMSC migration and osteogenic differentiation, enhance M2 macrophage polarization and reduce osteoclast differentiation in inflammatory diabetic defects (Song R. et al., 2025). These examples suggest that diabetic bone regeneration requires simultaneous regulation of glucose toxicity, redox imbalance, inflammation and vascularized osteogenesis.

In osteoporotic bone defects, regeneration is mainly limited by excessive osteoclast activity, insufficient osteogenesis and impaired bone homeostasis. HBC-nDBM-GelMA@ALN systems provide sustained alendronate and BMP-2 release, inhibiting osteoclastogenesis while promoting osteogenic activity (Lei et al., 2026). GelMA-QK/Sr-LDH@PDA hydrogels regulate osteoclast-lineage cells through Sr^2+^, preserve preosteoclast-associated PDGF-BB signaling and enhance H-type vessel-associated bone regeneration (He et al., 2025c). Macrophage membrane–liposome hybrid nanovesicles embedded in GelMA can scavenge RANKL, TNF-α and IL-6 while sequentially releasing PTH 1–34 to rebalance osteoclastic and osteoblastic activities (Li Q. et al., 2025). Other systems, including GA@GelMA microspheres, Ce@Mo/GC hydrogels and GelMA/Mg/DMF microspheres, improve osteoporotic repair by reducing senescence, oxidative stress, pyroptosis-related inflammation or abnormal macrophage activation (Hong et al., 2025; Lu et al., 2025; Song et al., 2025a; Wang R. et al., 2026). Together, these studies indicate that osteoporotic bone regeneration should be approached as the restoration of osteoimmune and bone-remodeling homeostasis, rather than simply as enhancement of bone formation.

Aging-related bone defects provide another example of immune-metabolic dysfunction. Inflammaging, senescent cell accumulation, impaired efferocytosis and reduced BMSC osteogenic potential can jointly weaken repair (Pignolo et al., 2021; Kushioka et al., 2023; Xu R. et al., 2023). GelMA hydrogels delivering grancalcin-neutralizing antibody target injury-induced grancalcin from senescent macrophages and improve aged jawbone healing by reducing macrophage-mediated impairment of BMSC mitochondrial function and osteogenesis (Dong et al., 2026). GA@GelMA microspheres and ROS-responsive FMAG nanoplatforms further alleviate cellular senescence, promote M2 macrophage polarization, enhance efferocytosis and improve periodontal tissue regeneration under aging-related inflammatory conditions (Hong et al., 2025; Wang Z. et al., 2025). These examples show that aging-related bone regeneration requires correction of immune senescence and stem-cell dysfunction in addition to conventional osteogenic induction.

Overall, the design logic of functionalized GelMA hydrogels in metabolic disorder- and aging-associated bone defects should shift from general pro-regenerative supplementation to disease-context-specific microenvironmental correction. Diabetic defects require coordinated regulation of hyperglycemia-driven oxidative stress, inflammation and vascular dysfunction; osteoporotic defects require restoration of osteoblast–osteoclast coupling; and aging-related defects require intervention in inflammaging, cellular senescence and macrophage–BMSC dysfunction. Therefore, these models should not be treated as interchangeable poor-healing conditions. Future studies need to define disease-specific mechanisms, match material functions with the dominant pathological barrier, and evaluate whether local osteoimmune modulation can support stable long-term bone remodeling. A further limitation is that GelMA-based interventions act predominantly at the local defect site and cannot by themselves correct systemic hyperglycemia, osteoporosis or aging-related dysregulation. The efficacy of local osteoimmunomodulation should therefore be interpreted in the context of concurrent control of the underlying systemic disease and evaluated using models that reproduce clinically relevant metabolic severity.

### 4.4 Oral and maxillofacial bone defects

Oral and maxillofacial bone defects differ from general bone defects because they are shaped by the oral microbiota, mucosal environment, periodontal ligament–alveolar bone interface, implant interface and masticatory loading. In periodontitis, peri-implantitis, medication-related osteonecrosis of the jaw (MRONJ) and radiation-induced jaw injury, infection or immune imbalance can further disrupt osteogenesis, osteoclast activity, angiogenesis and soft–hard tissue interface reconstruction. Therefore, functionalized GelMA hydrogels in this region should combine local retention, antibacterial activity, osteoimmune modulation, vascularization and structural adaptation.

Periodontal bone defects are among the most representative models because they involve both alveolar bone loss and disruption of the periodontal soft–hard tissue interface. Microneedle–nanosheet composite membranes provide temporally coordinated antibacterial, immunomodulatory, osteogenic and angiogenic regulation by releasing antimicrobial peptides from the GelMA layer and delivering MSC-derived exosomes from microneedles in rat and beagle periodontal defect models (Zhang X. et al., 2026). GelMA@MP196/exos promotes neutrophil apoptosis, macrophage efferocytosis and M2 macrophage polarization while inhibiting periodontal pathogens, thereby reducing alveolar bone loss (Zhuo et al., 2025). PGRN/GelMA used as an adjunct to scaling and root planing reduces M1 macrophage- and Th17-related markers and increases M2 macrophage- and Treg-related markers in a canine class II furcation involvement model (Liu W. et al., 2024). In addition, GelMA/dECM-based 3D-printed periodontal modules provide topographic cues and a dental follicle-derived matrix microenvironment, promoting alveolar bone mineralization, ordered periodontal fiber alignment and bone–ligament interface reconstruction (Yang et al., 2023). These studies suggest that periodontal regeneration should be evaluated not only by bone fill but also by restoration of the functional periodontal interface.

In mandibular, alveolar and craniofacial critical defects, morphological adaptation and spatial stability are particularly important. GelMA/DBM/USC-Exos 3D-printed scaffolds maintain exosome bioactivity and support sustained release, thereby promoting M2 macrophage polarization, angiogenesis and osteogenesis in mandibular defect models (Chen et al., 2026). Pomegranate-inspired conductive GelMA/PEDOT:PSS microgel/periosteum systems further buffer ROS, remodel macrophage metabolism and promote bone bridging in mandibular critical defects (Feng et al., 2026). For alveolar augmentation and guided bone regeneration, GelMA-stabilized Bio-Oss particles improve handling and space maintenance while promoting M2 macrophage responses, vascular ingrowth and new bone formation (Wang et al., 2023). Mechanically reinforced GelMA membranes and PCL-ACZP-GelMA biphasic modules further emphasize surgical operability, mechanical durability and alveolar morphology reconstruction (Wang S. et al., 2025; Sun et al., 2026). These studies indicate that oral clinical applications require bioactivity to be integrated with space maintenance, defect conformity and surgical handling.

Peri-implantitis is a distinct interface disease involving bacterial challenge, oxidative stress, chronic immune imbalance and peri-implant bone resorption (Berglundh et al., 2018; Huang et al., 2024). Chiral Fe_3_O_4_/GelMA hydrogels provide stage-adaptive regulation: D-FG alleviates acute oxidative stress through enhanced peroxidase-like activity, whereas L-FG promotes M2 macrophage polarization through the YTHDF1–m^6^A–Itgb3 axis and supports bone formation during chronic bone resorption (Shi et al., 2026). Sonoresponsive HMME@ZIF-8/Met/BMP-2@PLGA/GelMA systems further combine ultrasound-enhanced antibacterial activity with metformin and BMP-2 delivery to reduce inflammatory cytokine release and limit peri-implant bone loss (Hu B. et al., 2024). These examples indicate that peri-implantitis treatment requires simultaneous control of infection, immune stage transition and implant-associated bone regeneration.

MRONJ and radiation-induced jaw injury represent more complex oral pathological environments. GelMA/PD systems loaded with PTH and miR-218-transfected alveolar mucosa-derived stem cells improve soft tissue coverage, reduce sequestrum formation and restore osteoblast–osteoclast coupling in MRONJ-related wounds (Tu et al., 2025). Hep/GelMA-PRF scaffolds regulate PRF-derived growth factor release, reverse zoledronate-induced inhibition of osteogenic differentiation and alleviate local inflammation (Tao et al., 2024). In radiation-induced jaw injury, GelMA-encapsulated tolerogenic dendritic cells locally inhibit excessive dendritic-cell activation, reduce inflammation and promote bone regeneration (Zheng M. et al., 2025). These studies highlight the need for synchronized soft–hard tissue repair and precise osteoimmune regulation in special jaw injury models.

Overall, oral and maxillofacial defects impose anatomical and biological constraints that are not fully represented by conventional calvarial or long-bone models. GelMA offers particular practical advantages in this setting because its injectability, photocrosslinkability, adhesive or composite compatibility and printability can facilitate adaptation to irregular geometries and soft–hard tissue interfaces. These advantages, however, must be balanced against persistent oral microbial exposure, saliva and mucosal barriers, masticatory loading, limited local retention and implant-associated interfaces. Accordingly, functionalized GelMA systems for oral and maxillofacial regeneration should be evaluated not only by new bone formation, but also by infection control, mucosal compatibility, restoration of functional tissue interfaces, mechanical stability and clinically operable delivery. Future studies should therefore incorporate oral microbiome dynamics, masticatory mechanics, mucosal healing and long-term remodeling into indication-specific preclinical models.

## 5 Discussion and future perspectives

Although functionalized GelMA hydrogels have shown considerable promise for osteoimmunomodulation and bone regeneration, their clinical translation remains limited by gaps in manufacturing control, safety evaluation, mechanistic understanding and model validation. These barriers are closely linked to the increasing structural and functional complexity of functionalized GelMA-based systems.

Importantly, anti-inflammatory and antioxidant regulation should be regarded as upstream enabling events rather than regenerative end points. By relieving excessive ROS and persistent inflammatory signaling, these interventions can restore a microenvironment permissive for endothelial recovery, vascular maturation and subsequent osteogenesis. Accordingly, their regenerative efficacy should be evaluated by whether early immune/redox regulation ultimately propagates into functional vascularization and bone formation, as discussed mechanistically in Section 2 (Grosso et al., 2017; Li et al., 2021; Amersfoort et al., 2022; Gou et al., 2024).

The first major challenge is material standardization. The performance of GelMA hydrogels is highly sensitive to polymer characteristics and crosslinking conditions, which influence their mechanical behavior, degradation and biological activity (Yue et al., 2015; Huang et al., 2025). This variability becomes more difficult to control when inorganic nanocomponents, bioactive molecules, extracellular vesicles or blood-derived products are further incorporated. Therefore, future studies should establish quality-control parameters that link material composition and fabrication conditions to mechanical properties, degradation, cargo release and biological potency. Such standardization is particularly important for multifunctional systems, in which excessive functional integration may improve short-term experimental performance but also increase batch variability and translational uncertainty.

A related issue is the coupling between matrix degradation and cargo-release kinetics, which should not be assumed to be synonymous. In composite GelMA systems, release may reflect diffusion, protease-mediated matrix degradation, secondary-carrier disassembly or stimulus-triggered processes; thus, both overly rapid and overly slow kinetics can disrupt the timing of structural support, tissue infiltration and therapeutic availability. Future studies should therefore quantify matrix degradation and cargo release independently and relate both to stage-specific delivery requirements, including early immunoregulatory cues and later or sustained angiogenic/osteogenic signals (Man et al., 2022; Li et al., 2024d; Han C. et al., 2025; Yu C. et al., 2026).

Long-term safety also requires more systematic attention. Most current studies evaluate bone formation over relatively short observation periods, whereas the long-term *in vivo* fate of incorporated functional components remains insufficiently defined. This issue is particularly relevant for GelMA systems incorporating inorganic nanocomponents, nanozymes, nanosheets, metal–organic frameworks, carbon-based nanomaterials, extracellular vesicles or externally activated modules. Depending on the component class, relevant safety concerns may include local retention and clearance, degradation or transformation products, systemic biodistribution, immunogenicity, nanotoxicity and dose-dependent effects on osteoimmune homeostasis. These potential risks may not be fully captured by early histological or micro-computed tomography outcomes (Huang et al., 2025; Wang Y. et al., 2025; Shi et al., 2026). In addition, photocrosslinking-related residues and repeated external stimulation may introduce safety concerns that are specific to functionalized GelMA systems. Future evaluation should therefore extend beyond new bone volume and include chronic inflammation, component clearance, degradation–regeneration matching, systemic safety and the long-term stability of regenerated tissue.

Another important limitation is that many studies still describe osteoimmune regulation mainly through M1/M2 macrophage-associated markers. Although this framework is useful, it cannot fully explain the heterogeneous and dynamic immune responses induced by implanted materials. Single-cell and spatial omics approaches may help reveal how GelMA-based systems regulate immune-cell states, stromal-cell behavior and immune–vascular–osteogenic coupling within the defect niche (Yang X. et al., 2025). Future work should therefore move beyond phenotypic marker confirmation toward mechanistic validation and mechanism-guided material design.

Preclinical model selection remains another barrier to translation. Current evidence is still dominated by small-animal defect models, especially calvarial and femoral defects. These models are valuable for proof-of-concept studies, but they cannot fully reproduce human load-bearing conditions, remodeling kinetics or disease-specific immune environments. In oral and maxillofacial applications, models should also reflect periodontal pockets, mucosal barriers, oral microbiota, masticatory loading and implant-associated interfaces more closely. More standardized and stepwise validation, progressing from small-animal screening to large-animal and indication-specific models, is therefore needed to determine whether osteoimmunomodulatory effects observed in simplified settings can support stable bone regeneration under clinically relevant conditions.

Intelligent and personalized design may further expand the potential of functionalized GelMA hydrogels, but these strategies should be developed with clear translational boundaries. Machine learning and high-throughput experimentation could accelerate formulation optimization, while theranostic systems may allow non-invasive monitoring of scaffold degradation and local microenvironmental changes. Responsive platforms that convert pathological signals into therapeutic outputs, such as hyperglycemia-driven microcurrent generation for macrophage reprogramming (Lv et al., 2026), provide an example of closed-loop osteoimmunomodulation. Nevertheless, these advanced systems will only be clinically meaningful if their stimulation parameters, safety profiles and manufacturing processes can be reproducibly controlled. For craniofacial or periodontal defects, 3D printing and patient-specific scaffold fabrication and delivery strategies may improve anatomical adaptation, but bioink standardization and clinically compatible production remain unresolved (Varshney et al., 2025; Dixit et al., 2026).

Overall, the future development of functionalized GelMA hydrogels should shift from increasing functional complexity toward controllable, mechanism-defined and indication-specific design. The key issue is not how many components can be incorporated, but whether material composition, fabrication conditions, release kinetics, degradation behavior and stimulation parameters can be reproducibly aligned with the temporal sequence of inflammation resolution, vascularization, osteogenesis and bone remodeling.

## 6 Conclusion

Functionalized GelMA hydrogels have evolved from ECM-mimetic matrices and local delivery depots into programmable immunoregenerative platforms for osteoimmunomodulation and bone regeneration. Their regenerative value lies not simply in osteogenic performance or functional complexity, but in their ability to coordinate inflammation resolution, vascularization, osteogenesis and remodeling through the integrated regulation of matrix properties, functional cues and spatiotemporal activation. Across critical-sized, infected, metabolic disorder- and aging-associated, and oral and maxillofacial bone defects, effective GelMA-based designs should be adapted to the dominant pathological barrier rather than rely on generalized multifunctional integration. By linking material functionalization with osteoimmune-regenerative mechanisms and pathology-specific requirements, this review provides a design framework for the development of controllable and clinically relevant GelMA-based biomaterials. Their future translation will depend on reproducible manufacturing, mechanistic validation, long-term biosafety and evaluation in clinically relevant models.
